# Monitoring Wound Healing with Topically Applied Optical NanoFlare mRNA Nanosensors

**DOI:** 10.1002/advs.202104835

**Published:** 2022-04-22

**Authors:** Jangsun Hwang, Youngmin Seo, Daun Jeong, Xiaoyu Ning, Christian Wiraja, Lixia Yang, Chew Teng Tan, Jinhyuck Lee, Yesol Kim, Ji Won Kim, Dai Hyun Kim, Jonghoon Choi, Chin Yan Lim, Kanyi Pu, Woo Young Jang, Chenjie Xu

**Affiliations:** ^1^ School of Chemical and Biomedical Engineering Nanyang Technological University 62 Nanyang Drive Singapore 637457 Singapore; ^2^ Department of Orthopedic Surgery College of Medicine Korea University 73 Korea‐ro, Seongbuk‐gu Seoul 02841 Republic of Korea; ^3^ School of Electrical and Electronic Engineering Yonsei University 50 Yonsei‐ro, Seodaemun‐gu Seoul 03722 Republic of Korea; ^4^ Department of Research & Development OID Ltd 249‐2, 123 Osongsaengmyeong‐ro, Osong‐eup, Heungdeok‐gu, Cheongju‐si Chungcheongbuk‐do 28160 Republic of Korea; ^5^ NTU Institute for Health Technologies Interdisciplinary Graduate School Nanyang Technological University 61 Nanyang Drive Singapore 637335 Singapore; ^6^ A*STAR Skin Research Labs Agency for Science Technology and Research 8A Biomedical Grove Singapore 138648 Singapore; ^7^ School of Integrative Engineering Chung‐Ang University 84, Heukseok‐ro, Dongjak‐gu Seoul 06974 Republic of Korea; ^8^ Department of Dermatology College of Medicine Korea University 73 Korea‐ro, Seongbuk‐gu Seoul 02841 Republic of Korea; ^9^ Department of Biochemistry Yong Loo Lin School of Medicine National University of Singapore MD 7, 8 Medical Drive Singapore 117596 Singapore; ^10^ Department of Biomedical Engineering City University of Hong Kong 83 Tat Chee Avenue Kowloon Hong Kong SAR China

**Keywords:** diabetic wound, mRNA nanosensors, NanoFlare, spherical nucleic acids, wound healing

## Abstract

An effective wound management strategy needs accurate assessment of wound status throughout the whole healing process. This can be achieved by examining molecular biomarkers including proteins, DNAs, and RNAs. However, existing methods for quantifying these biomarkers such as immunohistochemistry and quantitative polymerase chain reaction are usually laborious, resource‐intensive, and disruptive. This article reports the development and utilization of mRNA nanosensors (i.e., NanoFlare) that are topically applied on cutaneous wounds to reveal the healing status through targeted and semi‐quantitative examination of the mRNA biomarkers in skin cells. In 2D and 3D in vitro models, the efficacy and efficiency of these nanosensors are demonstrated in revealing the dynamic changes of mRNA biomarkers for different stages of wound development. In mouse models, this platform permits the tracking and identification of wound healing stages and a normal and diabetic wound healing process by wound healing index in real time.

## Graphical abstract

This report describes the synthesis and utilization of mRNA nanosensors (i.e., NanoFlare) that are topically applied on cutaneous wounds to reveal the healing status through targeted and semi‐quantitative examination of the mRNA biomarkers. In both in vitro and mouse models, nanosensors reveal the dynamic changes of mRNA biomarkers for different stages of wound development.

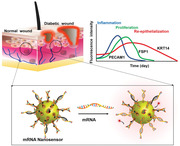

## 1 Introduction

Wound management is the modulation of inherent tissue capability to regenerate the damaged or lost skin tissue. Wound healing involves the spatial and temporal synchronization of different stages including homeostasis/coagulation, inflammation, proliferation/re‐epithelialization, and remodeling.^[^ ^1^ ^]^ Wounds that fail to proceed through an orderly and timely reparation to produce anatomic and functional integrity are considered as abnormal wounds.^[^ ^2^ ^]^ Diabetes and chronic infection are common causes of poor wound healing, among others. A successful wound management strategy requires the timely and accurate assessment of wound status for effective clinical decision making. Currently, this is done through simple visual observation of the wound or time‐consuming and disruptive methods like quantitative polymerase chain reaction (qPCR) to examine the specific metabolites/biomarkers in the wound fluid.^[^ ^3^ ^]^

Sensory nanomaterials or nanosensors rely on the unique physicochemical properties of nanomaterials to achieve medical diagnostics which are faster, cheaper, and more sensitive and accurate. Depending on the properties of the nanomaterials, the detection methods may be electrochemical, optical, or piezoelectric in nature. While they have widely been explored in the field of cancer diagnosis and prognosis, blood glucose monitoring, etc.,^[^ ^4^, ^5^ ^]^ their potential roles in wound assessment have not been widely explored. A few existing efforts target a specific metabolite/biomarker in the wound fluid,^[^ ^6^, ^7^, ^8^ ^]^ focusing on one stage of the wound healing process. However, as mentioned above, wound healing involves multiple stages with their unique biomarkers.^[^ ^3^, ^9^, ^10^ ^]^ A full picture of the wound healing process can only be revealed by a continuous and collective analysis of multiple biomarkers in different stages of wound healing.

**Scheme 1:**
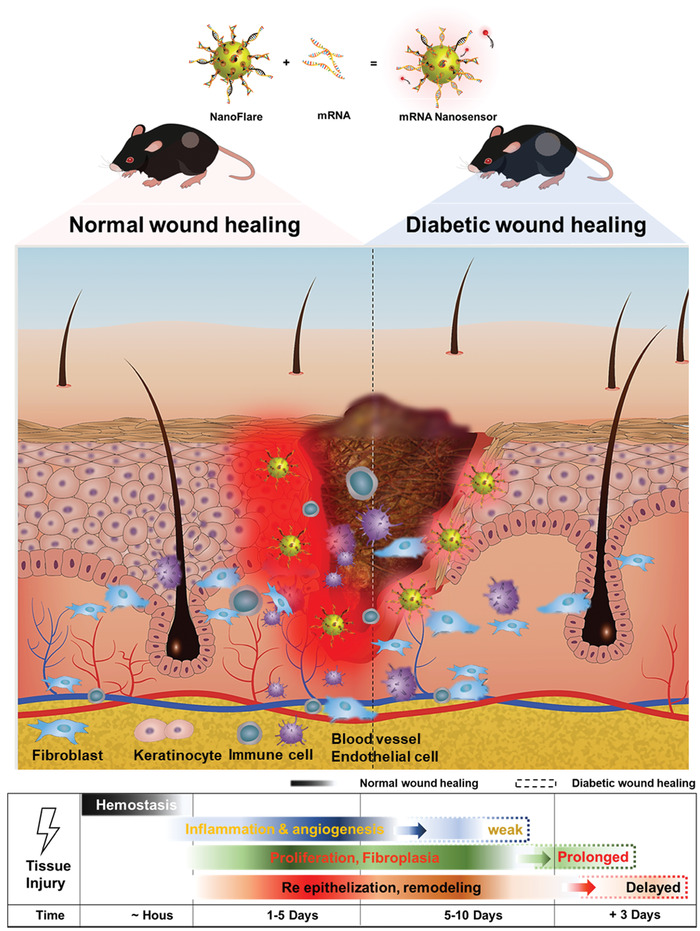
Illustration of NanoSensor (i.e., NFs) to detect cellular mRNA for monitoring wound status.

We recently reported the topically applied NanoFlares (NFs, a nanosensor for intracellular detection of mRNA based on Spherical Nucleic Acid [SNA] platform) for measuring the expression of connective tissue growth factor (CTGF) as a semi‐quantitative indicator of hypertrophic scars and keloids.^[^ ^11^ ^]^ These optical sensors can diffuse through skin layers, target and quantify cellular mRNA biomarkers, and report the information non‐invasively.^[^ ^12^ ^]^ Compared with the current visual observation and biopsy‐based methods, the optical methodology is more accurate, timely, convenient, and non‐invasive. Extrapolating from this report, we further hypothesize that NFs can be topically applied to monitor the status of the tissue and physiological changes of wound healing in real time (**Scheme** **1**). Specifically, we synthesize four kinds of NFs that identify stage‐specific and unique mRNA biomarkers which correspond to four stages of wound healing. Correlation between the changes of cellular environment and fluorescence signals from these NFs are established in vitro, and the ability of topically applied NFs to accurately identify normal or abnormal wound healing in vivo is assessed.

## 2 Results

### 2.1 Screening of RNA Biomarkers Related with Different Phases of Wound Healing

**Figure 1:**
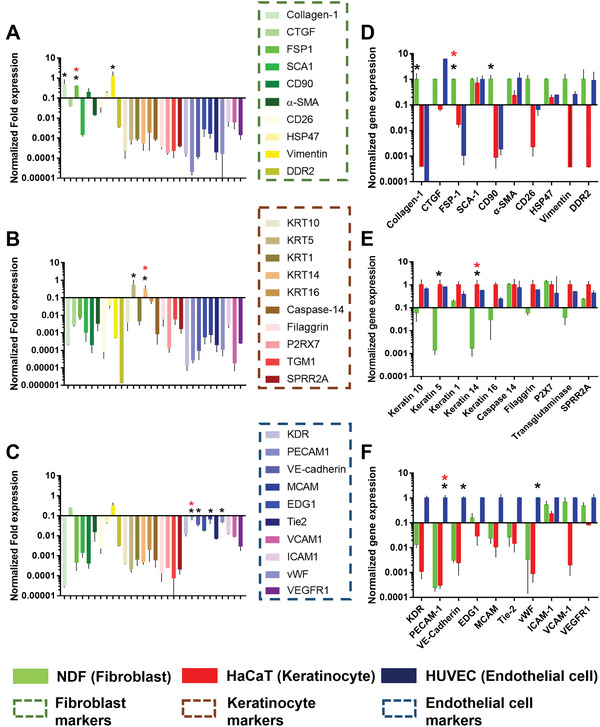
Screening of biomarkers for fibroblasts (NDF), keratinocytes (HaCaT), and endothelial cells (HUVEC). Cellular expression of the 30 potential RNA biomarkers in A) NDF; B) HaCaT; C) HUVEC. The cellular expression of 10 identified D) NDF biomarkers; E) HaCaT biomarkers; F) HUVEC biomarkers in NDF, HaCaT, and HUVEC respectively. Fold expression of each gene is normalized to the expression levels of housekeeping gene (GAPDH). The candidate gene is marked with a black star, selected gene is a red star respectively (n = 3, Values are means ± s.d.).

To identify the cell type‐specific mRNA biomarkers, we identified potential 10 mRNA biomarkers for three types of cells (i.e., endothelial cells, fibroblasts, and keratinocytes) from a pool of 153 candidates that have been reported.^[^ ^13^, ^14^, ^15^, ^16^, ^17^, ^18^ ^]^ Selection was based on the wide recognition and adaptation of these 10 genes in the literature. We then compared the cellular expression of these 10 mRNAs, identifying one candidate marker with strong expression in the target cell and weak expression in the other types of cells. **Figure** **1**A–C shows the expression of the 30 candidate mRNA biomarkers in each cell type, while Figure 1D–F reveals the relative expression of the 10 selected mRNA biomarkers in the target cells in comparison to the other two cell types.

As shown in Figure 1A,D, normal dermal fibroblasts (NDFs) strongly express fibroblast‐specific protein 1 (FSP1), Collagen‐1, and cluster of differentiation 90 (CD90) mRNA among the 30 potential biomarkers. FSP1 has previously been identified as a marker for fibroblasts in wound healing and fibrosis.^[^ ^14^, ^19^ ^]^ In addition, its expression was low in keratinocytes (HaCaT) and endothelial cells (HUVEC). Therefore, we chose FSP1 as the biomarker for fibroblasts.

Keratinocytes (HaCaT) showed strong expression of keratin 5 (KRT5) and keratin 14 (KRT14) mRNA (Figure 1B). KRT14 is specifically expressed in basal layer of epidermal keratinocytes and often used as a biomarker in the keratinocyte driven re‐epithelialization.^[^ ^20^, ^21^ ^]^ Here, we chose KRT14 as the biomarker for keratinocytes in wound healing. Note: each target gene, location, cell type, and description are presented as freely available in the Human Protein Atlas (www.proteinatlas.org). We noticed that KRT14 (0.56‐fold) expressions were only slightly lower in endothelial cells (HUVEC) than those in HaCaT^[^ ^22^ ^]^ (Figure 1E).

Endothelial cells (HUVEC) showed higher expression of platelet and endothelial cell adhesion molecule 1 (PECAM1) (i.e., CD31), kinase insert domain receptor (KDR), VE‐cadherin, and von Willebrand factor (vWF) (Figure 1C,F). PECAM1 regulates leukocyte migration and inflammatory, vascular responses and is also implicated in biological functions including angiogenesis, apoptosis, platelet aggregation, and thrombosis.^[^ ^23^, ^24^ ^]^ Therefore, PECAM1, FSP1, and KRT14 were chosen as the target biomarker genes for endothelial cells (inflammation, vascularization, proliferation), fibroblasts (fibroplasia, proliferation), and keratinocytes (proliferation, remodeling), respectively.

### 2.2 Design, Synthesis, and Characterization of NFs

After having identified the target genes corresponding to each stage of wound healing, we synthesized NFs targeting these mRNA biomarkers. NFs were composed of 13 nm gold nanoparticle (GNP) core and surrounding nucleic acid duplexes. The duplexes contain recognition sequences (termed as R) and the flare sequences (termed as F). We optimized the ratio of R/F by checking their complexing efficiency. As shown in Figure S1, Supporting Information, a higher ratio of R:F would provide higher hybridization. While the 10:1 ratio of R/F showed the highest R/F hybridization, we chose 5:1 R/F ratio for the subsequent synthesis to balance the hybridization efficiency with cost of assembly.

NFs were synthesized through a modified salt aging method^[^ ^25^ ^]^ that incorporated some features of the low temperature assembly method.^[^ ^26^ ^]^ This method provided the most sensitive NF that showed the biggest fluorescence signal change upon addition of target sequence (Figure S2A, Supporting Information). The clear absorption at 260 nm of NFs came from the nucleic acid duplexes on NFs (Figure S2B, Supporting Information). The assembly of R–F duplexes on GNP core also increased its hydrodynamic size from 13 to 28 nm (Figure S2C, Supporting Information) and decreased Zeta potential from −14 to −24 mV (Figure S2D, Supporting Information).

To optimize the sensitivity and stability of NFs, we studied the effect of flare sequence length by examining the fluorescence intensity change and Gibbs free energy change upon R–F hybridization (Table S1 and Figure S3, Supporting Information). Taking FSP1 NFs as the model, we changed the complementary flare sequence from 12 nucleotides to 18 nucleotides. The corresponding change of Gibbs free energy upon the hybridizations was calculated by RNA structure bifold software.^[^ ^27^ ^]^ As shown in Table S1, Supporting Information, a longer flare yielded a bigger change of Gibbs free energy, suggesting better R–F duplex stability. We further examined fluorescence restoration when the target sequence was added to solutions of FSP1‐NFs with different flare sequence lengths. As shown in Figure S4A, Supporting Information, all FSP1‐NFs except that with 12 nucleotides successfully restored its fluorescence signal. The fluorescence restoration was efficient and occurred within 2 min (Figure S4B, Supporting Information). As the fluorescence signal change was the biggest for FSP1 NFs with 15 nucleotide‐long flare (compared to 0.1, 4.3, 15, 9.6, and 4.1 times for 12, 14, 15, 16, and 18 nucleotide flare, respectively), alongside the lowest background signal, we chose 15 nucleotide‐long flare for subsequent synthesis of NFs.

**Figure 2:**
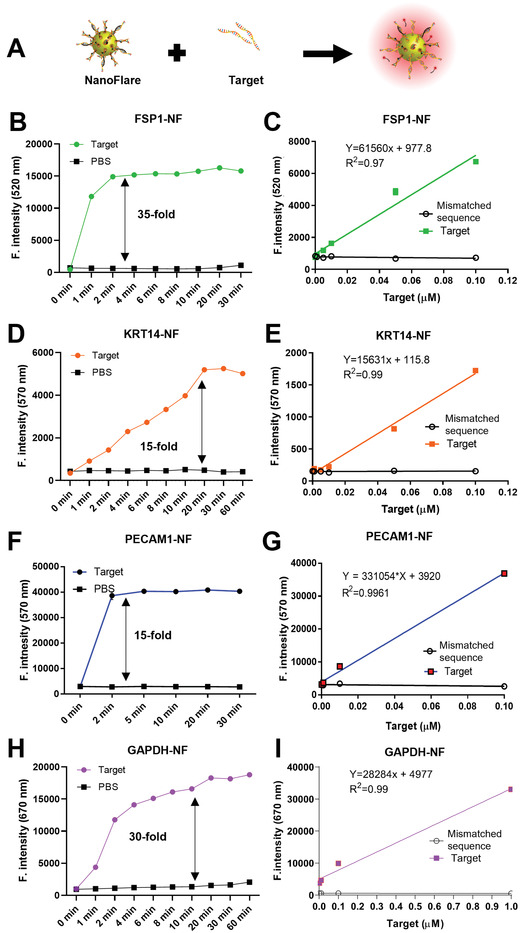
Identification of NF sensitivity. A) Illustrationof experiments; NF fluorescence restoration of B) FSP1‐NF, D) KRT14‐NF, F) PECAM1‐NF, and H) GAPDH‐NF over time after the mixing with concentration‐fixed target sequence and mismatched sequence (concentration of target sequence or mismatched target sequence is 2 µm; NF concentration is O.D 0.4, black line = PBS); Fluorescence restoration of C) FSP1‐NF, E) KRT14‐NF, G) PECAM1‐NF, and I) GAPDH‐NF after the mixing with varied concentrations of target sequence and mismatched sequence with a fixed incubation time (NF concentration is O.D 0.4, black line: mismatched sequence = 2 µm. Incubation time is 20 min, *n* = 3, values are means ± s.d. Excitation and emission of FSP1‐NF are 493 nm and 517 nm. Excitation and emission of KRT14‐NF and PECAM1‐NF are 555nm and 569 nm. Excitation and emission of GAPDH‐NF is 651 and 670 nm).

As mentioned above, PECAM1, FSP1, and KRT14 were chosen as the target biomarkers for endothelial cells (inflammation, vascularization, proliferation), fibroblasts (fibroplasia, proliferation), and keratinocytes (proliferation, remodeling). Therefore, we synthesized NFs that targeted these genes using the optimized synthesis protocol. NFs recognizing the glyceraldehyde 3‐phosphate dehydrogenase (GAPDH) reference gene were also synthesized with same protocol to act as control signal as well as cell quantification. All NFs showed good sensitivity to its respective target sequences (**Figure** **2**). For example, there was more than 35‐fold change of fluorescence intensity when FSP1‐NFs met the target sequence (Figure 2B ). Fluorescence signal showed a linear correlation with the concentration of target sequence at the range of ≈0.001–0.1 µm (*R* ^2^ = 0.97) (Figure 2C and Figure S5A, Supporting Information). Signal saturation was only observed when the target concentration was above 0.1 µm for GNP optical density (OD) of 0.4 (Figure S5A, Supporting Information). Similar results were obtained for KRT14‐NFs (Figure  2D,E and Figure S5B, Supporting Information, *R* ^2^ = 0.99). Meanwhile, PECAM1‐NFs (Figure  2F, G and Figure S5C, Supporting Information) and GAPDH‐NFs (Figure 2H, I and Figure S5D, Supporting Information) showed better sensitivity with target responsiveness between 0.0001 and 0.1 µm and good correlation (*R* ^2^ = 0.996 and 0.98, respectively). All NFs showed satisfactory selectivity, monitoring window, and specificity. As shown in Figure 2B,D,F,H, NFs only responded to their target sequence, although their signal recovery kinetics somewhat varied. The differences are expected due to the different binding energies between target and flare sequences. As we estimated in Table S2, Supporting Information, for human sequence (15 nt), the binding free energies (−Δ*G*) were FSP1‐NF (18.7 kcal mol^−1^), KRT14‐NF (20.8 kcal mol^−1^), PECAM1‐NF (19.4 kcal mol^−1^), and GAPDH‐NF (19.6 kcal mol^−1^). So KRT14 NF is most stable and followed by GAPDH NF. This echoes the slower response of KRT14 NF and GAPDH NF to the binding of target.

We further optimized NF working concentrations for intracellular experiments (Figure S6, Supporting Information), to facilitate subsequent usage in in vitro and in vivo models. There were reliable fluorescence changes for FSP1‐NF between OD ≈0.08–0.6, KRT14‐NF between OD ≈0.01–0.6, PECAM1‐NF between OD ≈0.01– 0.4, and GAPDH‐NF between ≈0.005–0.4 were detected. We then standardized the NF concentrations for subsequent experiments, that is, OD 0.1 for FSP1‐NF and 0.0125 for KRT14‐NF, PECAM1‐NF, and GAPDH‐NF (FSP1‐NF concentration was greater than others due to dye intensity). Additionally, cytotoxicity assay revealed that all NFs were non‐toxic up to the concentration of OD 2 (Figure S7A–D, Supporting Information).

### 2.3 Monitoring the Dynamic Change of Cellular mRNA Targets with NFs without GAPDH Normalization

To evaluate dynamic performance of the NFs, we stimulated NDF with transforming growth factor beta 1 (TGF*β*1) and basic fibroblast growth factor‐2 (FGF2),^[^ ^28^, ^29^ ^]^ HaCaT with TGF*β*1+EGF,^[^ ^30^ ^]^ and HUVEC with TGF*β*1+VEGF.^[^ ^31^, ^32^ ^]^ Subsequently, cells were labeled with relevant NFs and imaged. The fluorescence intensity was quantified at single cell level through normalization by Hoechst signal (i.e., NF signal/ Hoechst). In the case of NDFs (Figure S8A, Supporting Information), both TGF*β*1 and FGF2 treatment increased the normalized fluorescence signals of FSP1‐NF (2.2 ± 0.15‐fold and 1.33 ± 0.17‐fold, respectively) (Figure S8B, Supporting Information). qPCR analysis revealed the correlated upregulation of the FSP1 mRNA expression (41 ± 5‐fold and 9 ± 5‐fold by TGF*β*1 and FGF2 treatment, respectively) (Figure S8C, Supporting Information). We further examined the expression of FSP1 in different types of cells using FSP1‐NF (Figure S9A, Supporting Information). NDF showed the highest signal (3.1 ± 0.15‐fold) as compared to HUVEC which showed the lowest expression. The result matched well with qPCR results (Figure S9B,C, Supporting Information).^[^ ^33^ ^]^ The spherical nucleic acid (SNA)‐based NF is generally recognized by class A scavenger receptors and internalized via the endosomal pathway.^[^ ^34^, ^35^ ^]^ According to a previous study, SNAs were primarily localized in early endosomes after 1–2 h incubation with cells.^[^ ^36^ ^]^ Here, we confirmed that NFs were taken up by cells following the 2‐h incubation through endocytosis. As shown in Figure S10, Supporting Information, the signals from NFs overlapped with lysosomes.^[^ ^37^, ^38^ ^]^

We treated HaCaT cells with TGF*β*1 and epidermal growth factor (EGF) for assessment of KRT14‐NF. As shown in Figure S8D, Supporting Information, TGF*β*1 treatment did not bring any significant change of the KRT14 expression while EGF induced 50% decrease of KRT14 Figure S8E, Supporting Information). This was confirmed by qPCR analysis (Figure S8F, Supporting Information). We further examined the expression of KRT14 in other types of cells using KRT14‐NF and as expected HaCaT showed the highest expression (Figure S9D–F, Supporting Information, 4.3 ± 0.11‐fold).

PECAM1‐NF was finally used to track the influence of TGF*β*1 and vascular endothelial growth factor (VEGF) treatment in HUVEC (Figure S8G,H, Supporting Information). Fluorescence signal from cellular PECAM1‐NF was decreased by 20 ± 3% in the TGF*β*1 treated group but was increased by 30 ± 5% in the VEGF treated group. Consistent with FSP1‐NF and KRT14‐NF, qPCR analysis verified this observation (Figure S8I, Supporting Information). PECAM1‐NF was also used to detect levels of PECAM1 expression in NDF, HaCaT and HUVEC (Figure S9G, Supporting Information). These experiments showed PECAM1‐NF exhibited the highest signals in HUVECs (1.45 ± 0.01‐fold) which corresponded to gene expression patterns detected by qPCR (Figure S9H,I, Supporting Information).

Taken together, NFs allowed the monitoring of dynamic cellular mRNAs, even without signal normalization against a reference gene.

### 2.4 Growth Factors Screening Using NFs for Monitoring Target Genes with GAPDH Normalization

**Figure 3:**
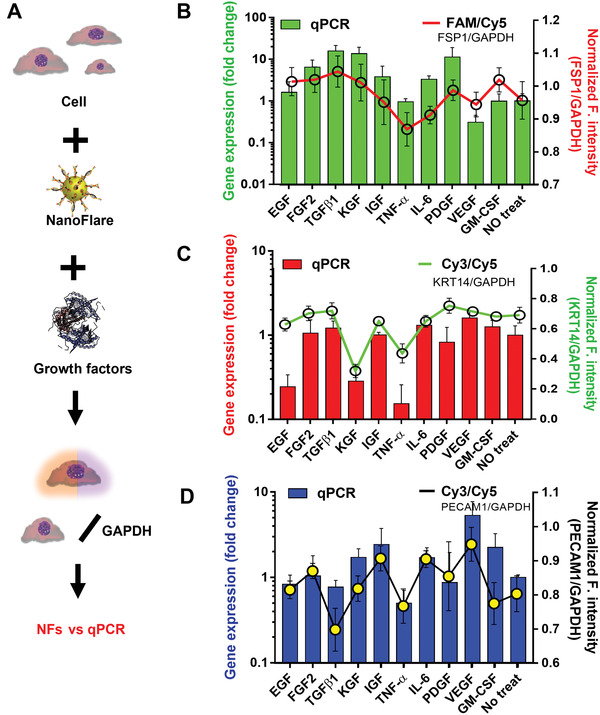
Evaluation and confirmation of target gene expression under the growth factor stimulation by NFs and qPCR. A) Illustration; B) FSP1 expression in NDF treated with 10 growth factors. The results were derived from FSP1‐NF signal (Figure S11, Supporting Information, red line plot, *n* = 11) and qPCR (green bar plot); C) KRT14 expression in HaCaT treated with 10 growth factors. The results were derived from KRT14‐NF signal (Figure S12, Supporting Information, green line plot, *n* = 10) and qPCR (red bar plot); D) PECAM1 expression in HUVEC treated with 10 growth factors. The results were derived from PECAM1‐NF signal (Figure S13, Supporting Information, black line plot, *n* = 6) and qPCR (blue bar plot). The signals of FSP1‐NFs, KRT14‐NFs, and PECAM1‐NFs were normalized using that of GAPDH‐NFs, values are means ± s.d.

We further explored the capabilities of NFs to analyze the stimulation effect of growth factors which have unclear effects on the three types of cells (**Figure** **3**A). We chose 10 growth factors that were known to be involved in wound healing.^[^ ^39^, ^40^ ^]^ These include EGF, FGF2, insulin‐like growth factor (IGF), keratinocyte Growth Factor (KGF), platelet derived growth factor (PDGF), TGF*β*1, VEGF, granulocyte macrophage colony‐stimulating factor (GM‐CSF), Interleukin 6 (IL‐6), and tumor necrosis factor alpha (TNF‐*α*). Here, GAPDH mRNA was taken as the reference gene and monitored for qPCR comparison.^[^ ^11^ ^]^

In fibroblasts (NDF), the FSP1 expression was elevated by TGF*β*1 (8%), FGF2 (7%), KGF (7%), PDGF (3%), GM‐CSF (7%), and EGF (5%), while IGF and VEGF did not induce significant changes. Conversely, TNF‐*α* and IL‐6 reduced FSP1 expression by 10% and 4%, respectively (Figure 3B, red line, and Figure S11, Supporting Information). qPCR analysis confirmed these results where FSP1 expression was the highest after TGF*β*1 treatment and lowest after VEGF and TNF‐*α* treatment (Figure 3B, green bars).

In keratinocytes (HaCaT), qPCR analyses showed that there was no change in KRT14 expression following the treatment with FGF2, TGF*β*1, IGF, and GM‐CSF. However, IL‐6 slightly increased KRT14 expression while EGF (0.24‐fold), KGF (0.28‐fold), and TNF‐*α* (0.15‐fold) down‐regulated KRT14 expression (Figure 3C, red bars). We observed no changes post the treatment with FGF2, TGF*β*1, IGF, VEGF, and GM‐CSF, with NF signals decreased in the samples treated with EGF (8%), KGF (54%), and TNF‐*α* (37%) (Figure 3C, green line, and Figure S12, Supporting Information). Hence, the qPCR and NF results were highly concordant.

In endothelial cells (HUVEC), the fluorescence signal from PECAM1‐NF increased after the treatment of FGF2, IGF, IL‐6, and VEGF, while TGF*β*1, TNF‐*α*, and GM‐CSF down‐regulated PECAM1 signals (Figure 3D, black line, and Figure S13, Supporting Information). qPCR confirmed that TGF*β*1 and TNF‐*α* decreased while KGF, IGF, IL‐6, and VEGF increased the PECAM1 expression (Figure 3D, blue bars).

Together, these results confirm the efficacy of NFs in monitoring the dynamic changes of target gene in different types of skin‐related cells.

### 2.5 NF Penetration and Specific Labeling of Cells in 3D Cell Spheroids

**Figure 4:**
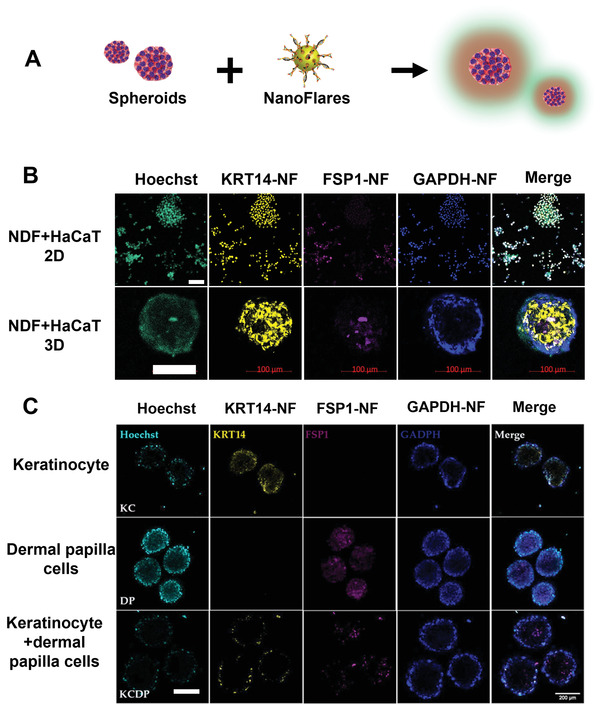
Confocal fluorescence images of 3D spheroid and 2D cell culture with NFs. A) Illustration of experiments; B) 2D and 3D co‐culture of NDF and HaCaT with NFs (white scale bar is 50 µm); and C) KC with NFs: top, DP with NFs: middle, co‐culture of KC and DP with NFs; bottom (scale bar is 200 µm). Concentrations of FSP1‐NF, KRT14‐NF, and GAPDH‐NF were O.D 0.1, 0.0125, and 0.0125, respectively.

NFs were further tested in 2D and 3D co‐culture models (**Figure** **4**A). We co‐cultured NDF and HaCaT using different strategies and created 2D and 3D models. As shown in Figure 4B, NFs introduced into both models could successfully identify the target genes in specific cells. For example, FSP1‐NF fluorescence was only seen in NDF while KRT14‐NF signal was visible only in HaCaT cells. GAPDH‐NF signal (reference signal) was seen in both types of cells.

We also examined whether the NFs could detect the change of gene expression in the 3D culture. HaCaT and NDF spheroids were first treated with TGFβ Receptor 1 (TGFBR1) small interfering RNA (siRNAs), TGFBR1 siRNAs +TGF*β*1, or TGF*β*1 prior to incubation with KRT14‐NF/GAPDH‐NF and FSP1‐NF/GAPDH‐NF, respectively (Figure S14, Supporting Information). There were 27% and 7% reduction of FSP1‐NF signals in spheroids treated with TGFBR1 siRNAs and siRNA+TGF*β*1, respectively, though the decrease was not statistically significant in TGF*β*1 treated group (Figure S14A,C,E, Supporting Information). KRT14‐NF signals remained unchanged in the HaCaT spheroids under all conditions, which is consistent with the qPCR results (Figure S14B,D,E, Supporting Information). This revealed that gene‐specific NFs could detect fluctuations in gene expression in the 3D cultures upon perturbation in TGF*β* signaling and support the further development of gene‐specific NFs to track gene expression changes directly in cells or tissues under physiological conditions.

To confirm the broad applicability of our NFs and the optimized methodology, we tested the NFs on the 3D cell spheroids prepared using keratinocytes and fibroblasts from other sources (Figure 4C). 3D cell spheroids were prepared from human epidermal keratinocytes (KCs), human dermal papilla cells (DPCs), or co‐cultured KCs and DPCs (KCDP). The KC‐, DPC‐, and KCDP‐spheroids with ≈0.01 mm^3^ dimensions were incubated with a cocktail of KRT14‐NF, FSP1‐NF, and GAPDH‐NF for 16 h and then assessed by confocal fluorescence imaging. The KRT14‐NF signal was specifically detected in KC‐ and KCDP‐spheroids, while the FSP1 signal was observed in DP and KCDP spheres (Figure 4C). We noted that while the fluorescence signal of GAPDH‐NF was detected in all cells, those of KRT14‐NF and FSP1‐NF did not overlap in the KCDP co‐culture spheroids. All these findings suggest that the NFs effectively penetrated into cells within the 3D spheroids and labeled cells with high specificity.

### 2.6 Monitoring Wound Healing in the Normal and Diabetic Mice

**Figure 5:**
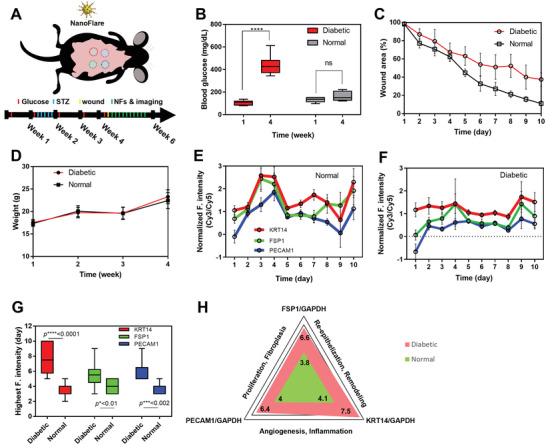
Monitoring wound healing by topically applied NFs on normal and diabetic mice. A) Illustration of experiment setup; B) blood glucose level, C) wound area, and D) weight change in normal and diabetic mice through the experiments; Evaluation of three biomarkers by NFs on, values are means ± s.d. E) Normal mice group and F) diabetic mice group (the signal was normalized against that of reference gene, GAPDH, means ± s.d); G) comparison of the date when the highest NF fluorescence signal was observed for each biomarker in two wound healing models from (E) and (F) (*p < 0.05, ** *p* < 0.01, *** *p* < 0.001, **** *p* < 0.0001, and ns = not significant, *n* = 6, duplicate); and H) Fluorescence Wound healing Index for normal and diabetic groups (mean value of the highest fluorescence signal from NF was normalized with GAPDH signal in 10 days in both groups respectively, *n* = 11, values are means ± s.d.).

We have confirmed that the designed NFs could reveal the target genes within both 2D and 3D dynamic environments. Next was to determine whether NFs would perform similarly in vivo. Specifically, we used NFs to study and compare wound healing in normal and diabetic mice models. Each of NF (GAPDH‐Cy5, PECAM1‐Cy3, KRT14‐Cy3, and FSP1‐Cy3 NFs) was first mixed with moisturizer and then topically applied on wounds individually 4 h after wound creation (**Figure** **5**A). In vivo fluorescence imaging was subsequently performed for 10 consecutive days (NFs were re‐applied 1 h after imaging from day 2 to 9, Figure S15A, Supporting Information). Diabetic mice showed consistently elevated blood glucose level (440 ± 87 mg dL^−1^) than normal mice (107 ± 20 mg dL^−1^) throughout the experiment (Figure 5B). Wounds healed faster in normal mice than diabetic mice (Figure 5C) while there was no significant difference of weight changes in both mouse groups (Figure 5D). The collected NF fluorescence signals (Figure 5E,F) were quantified by ImageJ where the mean fluorescence value was derived by subtracting the background signal from the fluorescence signals.

In normal wounds, FSP1 signal was observable from day 2, peaked at day 5, and then decreased afterwards (Figure S15B, Supporting Information). Differently, FSP1 signal continuously increased at day 5 and remained until day 9 on diabetic wounds. The profile of KRT14 signal was similar to that of FSP1 on normal wounds, where KRT14 signal appeared from day 2 and peaked at day 5 (Figure S15C, Supporting Information). However, KRT14 signal appeared at day 2 and reached the peak at day 8 on diabetic ones. Notably, for both types of wounds, the KRT14‐NF signal decreased significantly slower after the peak as compared to FSP1‐NF signal. On the other hand, PECAM1‐NF signal was found from day 2 to 7 and reached the peak at day 5 for the normal group (Figure S15D, Supporting Information). Diabetic group displayed similar fluctuation, but the peak was shifted to the right (occurred at later timepoint). In addition, fluorescence signal from the reference gene, GAPDH mRNA was monitored (Figure S15E, Supporting Information).

We normalized the FSP1, KRT14, and PECAM1 signals against the reference gene GAPDH signal (Figure 5E,F). On the normal wounds, all normalized three signals showed similar fluctuation and peaked at day ≈2–4 and day 10 (Figure 5E). However, on the diabetic wounds, there was no significant increase until day 8 (Figure 5F). There were 2–3 days delay for the appearance of maximal fluorescent signal (Figure 5G).

Based on the fluorescence peaks of each NF in Figure 5G, we established a wound healing diagram (Figure 5H). The Fluorescence Wound healing Index (FWI) can be derived by comparing the maximal fluorescence of each NF in the diabetic group to that of control group. Specifically, the maximal fluorescence signals for normal wound were FSP1 (3.8), KRT14 (4.1), and PECAM1^[^ ^4^ ^]^ while diabetic ones showed FSP1 (6.5), KRT14 (7.5), and PECAM1 (6.4). Therefore, FWIs were 1.7 for fibroplasia‐proliferation (FSP1), 1.9 for re‐epithelialization‐remodeling (KRT14), and 1.6 for angiogenesis‐inflammation (PECAM1). The value of FWIs indicates the deviation of diabetic wound healing from normal one.

Collectively, NF‐derived signals confirmed that diabetic wound exhibited delayed wound closure, prolonged inflammation, poor angiogenesis, and less matrix deposition in wound bed as compared to the normal wound healing.^[^ ^41^ ^]^

## 3 Discussion

Wound healing involves the spatial and temporal synchronization of a variety of cell types in different stages: homeostasis/coagulation (platelets, immune cells), inflammation (immune cells and endothelial cells), proliferation (fibroblasts and endothelial cells), and remodeling (keratinocytes). Homeostasis takes precedent at the earliest stage to stop the bleeding. It usually takes a few minutes and might last 1–2 days in certain extreme cases. It is impossible to apply NFs to monitor such swift processes and thus this study focuses on the later three phases that last at least a few days. The inflammatory phase is marked by platelet accumulation, coagulation, and leukocyte migration. Angiogenesis is also initiated in this stage immediately after tissue injury. Timely transition from the inflammatory stage to proliferative stage is a key step during normal wound healing, where fibroblasts play a critical role such as potent protagonists of fibroblast survival, migration, and metabolism. Later, re‐epithelialization occurs and involves interactions among keratinocytes and fibroblasts.

Wound healing is a complex series of reactions and interactions among cells, identification of each wound healing stage is impossible. This study aims at identifying the wound healing pattern by NF's signal. To achieve this goal, we chose three biomarkers (i.e., PECAM1 for endothelial cells, FSP1 for fibroblasts, and KRT14 for keratinocytes) reflecting different process of wound healing (inflammation‐ vascularization‐proliferation, fibroplasia‐ proliferation, and proliferation‐remodeling). They were identified from a pool of 153 related biomarker candidates that have been reported relevant to skin wound healing (Figure 1). Their expression in each type of cells is unique and a stronger expression is expected to indicate the increased cell proliferation. Accordingly, we synthesized four types of NFs that recognized and quantified three biomarkers and one reference gene through the modified salt aging method. This updated method shortened the synthesis time by at least a day as well as providing better target specificity (Figure S2A, Supporting Information). In both 2D and 3D cell experiments, NFs synthesized for these biomarkers accurately revealed the different expression of three RNA biomarkers in the associated cell types (Figures 3 and 4) and their changes caused by growth factors and siRNA treatment (Figure 3 and Figure S14, Supporting Information). One feature of these NFs is the good correlation of fluorescent signals with the target concentration range of ≈0.001–0.1 µm in the solution (Figure 2 and Figure S5, Supporting Information). While the precise intracellular concentrations of PECAM1, FSP1 and KRT14 mRNAs were not identified, GAPDH mRNA was estimated to have ≈1000 transcript copies per single‐cell equivalent.^[^ ^42^ ^]^ Taking the average cell volume as 4 × 10^−12^ L, the GAPDH mRNA cellular concentration would be ≈0.0004 µm. While this seems to fall below the detection range identified through in solution target sequence hybridization, it is noteworthy that these target mRNAs are typically localized within certain sections of cells (e.g., nucleus and ribosomes), therefore likely having significantly greater local cellular mRNA concentration than calculated here.

These NFs were finally used to track the wound healing in both normal and diabetic mouse models (Figure 5 and Figure S15, Supporting Information). Different from our previous work in scar identification,^[^ ^11^ ^]^ NFs were here directly applied on the wound tissue on a daily basis. NFs were re‐applied 1 h after imaging from days 1 to 9. This new protocol was to serve the dynamic changes in the wound healing process. Fluorescence signals from the three biomarkers were normalized against the reference gene signal (i.e., GAPDH mRNA). Clearly, the changes of biomarker‐related fluorescence signals were different in normal and diabetic mice. In the normal wound healing setting (Figure 5E), FSP1, KRT14, and PECAM1 signals showed similar fluctuation with maximal expressions at day ≈2–4. This observation is comparable to previous reports. Saraswati et al found that FSP1^+^ stromal cells were detectable at day 4 in skin.^[^ ^43^ ^]^ Qiang et al. found that at day 2 post‐wounding, KRT14‐positive keratinocytes increased along the wound edge and migrated into the wound in the mouse model.^[^ ^44^ ^]^ Etich et al. saw the proportion of PECAM1^+^ cells increased after day 3.^[^ ^45^ ^]^ In contrast, the expressions of the three biomarkers were lower for the diabetic mice group, especially in the first 5 days (Figure 5F). There was an average of 3 ± 0.7‐day delay for the appearance of fluorescence intensity peaks (Figure 5G). This observation echoes previous reports as well. Lerman et al. showed the impaired migration of diabetic fibroblasts (FSP1^+^).^[^ ^46^ ^]^ Terashi et al. and Aoki et al. showed that there was inhibited keratinocyte migration and proliferation and reduced KRT14 expression in diabetic wounds.^[^ ^47^, ^48^ ^]^ Tang et al. reported the inhibited PECAM1 expression with high glucose in 2D cell culture model.^[^ ^49^ ^]^ Ultimately, these results allowed the generation of the FWI graph (Figure 5H), which showed the deviation of diabetic wound healing from the normal one in terms of delayed wound closure, prolonged inflammation, poor angiogenesis, and less proliferation/re‐epithelialization.

In addition, we would highlight that this study was not designed for simultaneous detection of multiple biomarkers in the same wound. As wound healing stage is distinguishable in clinical cases especially patients with diabetic foot ulcer (DFU), there is distinctive morphological difference in DFU for inflammation to proliferation stage, and proliferation to re‐epithelialization stages (Figure S16A–D, Supporting Information). Specific NF will be selected for topical application upon wound status. For example, PECAM1‐NF enables to reveal inflammation stage with ischemic wound. Progression of inflammation to proliferation stage can be monitored by FSP1‐NF. KRT14 should be checked when the epithelialization stage is postponed in patients with DFU. In a clinical point of view, DFU treatment is open performed by subjective algorithm.^[^ ^50^ ^]^ If a wound healing stage would be revealed using a minimal invasive method, this would provide the clear and objective evidence to decision makers for treating DFU in a timely manner. Thus, an alternative procedure based on patient's condition with FWI of each stage provides better algorithm (Figure 5H and Figure S16E,F, Supporting Information). For instance, if PECAM1 signal shows lower in FWI without any inflammation symptom displaying an ischemic wound, anti‐thrombotic or low‐energy extracorporeal shockwave therapy may be applied in this case. When the wound shows delayed proliferation with lower FSP1 signal in FWI, topical use of EGF should be considered . In addition, if there is lower KRT14 signal in FWI (suggesting the lack of re‐epithelialization), skin graft is an alternative procedure to patients with DFU.^[^ ^50^ ^]^

Noteworthily, there are several limitations of this study. Although we screened and picked the best biomarker for each type of cells, these biomarkers are also expressed to a certain degree in other types of cells in vivo. For example, KRT14 is also highly expressed in endothelial cells (Figure 1). This prevents us to differentiate different status of wound healing strictly through the signals of these biomarkers. In addition, considering that each NF was individually applied on one wound site and each wound could undergo different wound healing rate, the comparison of three biomarker signal profiles is significantly influenced by variations between individual wound. Finally, while the handheld fluorescence microscopy is available, we still use in vivo imaging system (IVIS) for this study to standardize the operation for consistency with our previous studies. A similar study but using handheld fluorescence microscopy is under way to facilitate the translation of this methodology to clinical settings. Finally, qPCR analysis is lacking from our in vivo experiments to support our observations from NF‐mediated in vivo imaging. This is due to the disruption of our animal experiments by the pandemic management, which we hope to perform through our subsequent work. Nonetheless, our observed signal profiles are agreeable with previous reports, suggesting the promising potential of NF to facilitate facile and biomarker‐based wound healing monitoring.

## 4 Conclusion

This article reports the development and utilization of mRNA nanosensors (i.e., NFs) that were topically applied and revealed the wound status through targeting and quantifying the mRNA biomarkers in skin cells. The NF's efficacy and efficiency in revealing the dynamic changes of mRNA biomarkers for different stages of wound development were demonstrated in 2D and 3D in vitro and in vivo models. In mouse models, this platform permited the tracking and identification of wound healing stages and normal and diabetic wound healing process by FWI in real time. In future, we plan to repeat this methodology using handheld fluorescence microscopy for potential translation into clinics. In addition, besides surgical wounds, burn wounds will be examined in future studies.

## 5 Experimental Section

Streptozotocin (STZ) and growth factors (EGF, FGF2, TGF*β*1, KGF, IGF, TNF‐*α*, IL‐6, PDGF, VEGF, and GM‐CSF) were purchased from Sigma Aldrich (USA). PureLink RNA Mini Kit, tris(2‐carboxyethyl) phosphine (TCEP), and Matrigel were purchased from (Thermo Fisher Scientific). 13 nm citrate‐capped GNPs were synthesized as described previously.^[^ ^11^ ^]^ All DNA oligos were purchased from Integrated DNA Technologies with HPLC purifications. Other chemicals and agents were all purchased from Sigma Aldrich (USA) except where specifically mentioned.

### Cell Maintenance

NDFs, Human keratinocytes (HaCaT), and HUVEC were purchased from Cell Research Corporation (Singapore) and used between passages 3 and 10. HUVEC were maintained with Endothelial Cell Growth Medium‐2 Bullet Kit (Lonza). All other cells were maintained with DMEM. All the media were supplemented with 1% antibiotics (penicillin 100 U mL^−1^ and streptomycin 100 µg mL^−1^) and heat inactivated 10% fetal bovine serum (FBS) in 5% CO_2_ at 37 °C.

### qPCR Analysis for Selection of Target Sequences

Cells (NDF, HaCaT, and HUVEC) were seeded on the 6‐well plate without any stimulation at a density of 2 × 10^4^ and cultured to reach the confluency of 80%. Cells were then harvested for the extraction of total RNA using PureLink RNA Mini Kit (Thermo Fisher Scientific). 1 µg RNA was reverse transcribed using qScript cDNA SuperMix (Quanta BioSciences). Real‐time qPCR was performed on the cDNA with Light‐Cycler480 SYBR Green I Master on a CFX96 Touch System (Bio‐Rad). Primers were purchased from Integrated DNA Technologies (Table S3, Supporting Information). mRNA expression was normalized to the GAPDH expression using the 2^−∆∆Ct^ method. Each experiment was performed three times in duplicate. For the growth factor stimulation study, cells were first starved for 24 h in DMEM with 1% FBS before the addition of growth factors for 2 days.

### NFs Design and Synthesis

Table S4, Supporting Information, lists the thiol‐modified recognition sequences (termed as R) and the flare sequences (termed as F) that were modified with fluorescent dyes at 5′ end. Recognition sequences are complementary to the target sequences. To optimize sensitivity and stability of NFs, we set the predicted free energy change of all R–F duplexes was >−20 kcal mol^−1^ and R–T (Recognition–Target) was <‐60 kcal mol^−1^ (Table S1, Supporting Information).

Recognition strands (100 µm) and flare strands (100 µm) were annealed from 95 °C to room temperature (RT) over 1 h in different molar ratios (1:1, 2:1, 5:1, and 10:1, v/v) (Figure S1, Supporting Information). The resulted R–F duplexes were treated with 50 mm TCEP at RT for 1 h to activate the thiol‐modified recognition strand before being purified with Micro Bio‐Spin 6 Columns (Bio‐Rad). The purified R‐T duplexes were added to 13 nm citrate‐capped GNPs (1 mL, 5 nm). Next, the mixture was stored at −20 °C for 2 h, followed by adding 50 µL of 1 m NaCl every 30 min until the final NaCl concentration reached 0.3 m.^[^ ^26^ ^]^ The solution was sonicated for 30 s after each addition of NaCl. After being stirred overnight, functionalized GNPs or NFs were derived after centrifugation at 14 000 g for 30 min. NFs were washed twice with 0.05% Tween‐20 in PBS. Their concentrations were determined by the absorbance at 520 nm (Nanodrop 2000, Thermo Fisher Scientific). Hydrodynamic size and zeta potential of SNAs were measured using a dynamic light‐scattering system (Zetasizer, Malvern, UK).

### Cytotoxicity Test

HaCaT, NDFs, and HUVEC were cultured in 96 well plate at the confluency at 80% and then treated with different concentrations of particles (OD = maximum absorbance at 520 nm: value of 0, 0.05, 0.15, 0.25, and 0.4 for both GNPs and NFs to 0,0.1, 0.5, 1, and 2 for all NFs) for 24 h. Next, 100 µL of 1:20 diluted Cell Counting Kit‐8 (CCK‐8) solution (CCK‐8: DMEM, v/v) was added to each well and incubated for 4 h. The percentage of live and dead cells was spectrophotometrically analyzed at 450 nm.

### Hybridization of NFs with Free Target Strand

50 µL of NFs at the OD of 0.4 were mixed with target strands at 37 °C for 10 min in the 96‐well plate. The fluorescence intensities were examined at 520 nm for FSP1‐FAM NF, 570 nm for KRT14‐Cy3 NF, 570 nm for PECAM1‐Cy3 NF, and 670 nm for GAPDH‐Cy5 NF using Synergy HT Microplate Reader (BioTek). The concentrations of target strand were ranged from 0 to 10 µm to evaluate the sensitivity of NFs. Each measurement was repeated three times in duplicate.

### Hybridization of NFs with Target RNA in the Cellular RNA Extract

RNA extraction from 2D cultured cells was carried using PureLink RNA Mini Kit (Thermo Fisher Scientific). Then NFs were mixed with the total cellular RNA extract at RT for 30 min. The fluorescence intensities of the mixture were examined using Synergy HT Microplate Reader (BioTek). Each measurement was repeated three times in duplicate.

### NFs for Quantifying Cellular Expression of Target Genes

Cells on 48‐well plate were starved for 24 h in DMEM with 1% FBS. Later FGF2/TGF*β*1, EGF/TGF*β*1, and VEGF/TGF*β*1 were added to the media for NDF, HaCaTs, and HUVEC, respectively. 2 days later, the cells were washed with PBS and fed with 5% FBS medium containing NFs (the concentrations were OD 0.125 and 0.1). 16 h later, cells were stained with Hoechst 33 342 and subjected to confocal imaging (laser scanning confocal microscopy [LSCM], Carl Zeiss). Additionally, total RNA was extracted from the same experimental groups, and then treated with NFs before the measurement of fluorescence intensity. qPCR was also performed with the extracted RNA to compare with results from NF quantification. The comparison of target gene expression in NDF, HaCaT, and HUVEC were carried in a similar way but without stimulation.

### NFs for Identifying the Potent Growth Factors

Cells were cultured in the 48‐well plate and starved for 24 h before individual growth factor was added (EGF: 40 ng mL^−1^, FGF2: 40 ng mL^−1^, TGF*β*1: 40 ng mL^−1^, KGF: 40 ng mL^−1^, IGF:50 ng mL^−1^, TNF‐*α*: 40 ng mL^−1^, IL‐6: 40 ng mL^−1^, PDGF: 8 ng mL^−1^, VEGF: 20 ng mL^−1^, and GM‐CSF: 20 ng mL^−1^ in 1% FBS/DMEM) and incubated for 48 h. Next, DMEM containing OD 0.1 FSP1‐NF, OD 0.0125 KRT14‐NF, PECAM1‐NF, and GAPDH‐NF were added for another 16 h. After washing, 1 mL of Hoechst 33 342 working solution was added for 30 min to stain the cell nuclei. Cells were washed with PBS two times and imaged under confocal microscope (Carl Zeiss). The laser intensity and exposure were kept constant among experiments, which were performed in RT with minimized background light.

### Spheroid Formation and NF Penetration Assay

Immortalized normal human keratinocytes (KCs, N/TERT‐1, courtesy of J. G. Rheinwald) and human primary hair follicle DPCs (Adult, Cell Application Inc.) were used to generate the 3D spheroid cultures. KCs and DPCs were grown in Keratinocyte serum‐free media (KSFM) and DMEM supplemented with 10% fetal bovine serum (Hyclone) and 0.14 mg mL^−1^ bone pituitary extract (Gibco), respectively. Single cell suspensions were seeded into low attachment 96‐well plates (Corning) and incubated at 37 °C for spheroid formation.

To generate spheroids, 2000 KCs, 2000 DPCs and 2000 KCs + 2000 DPCs were seeded into each well to generate KC, DP, and the KCDP spheres. The spheroids were collected at day 3 post‐seeding and incubated in 100 µL of 1% FBS DMEM containing a mixture of KRT14‐NF, FSP1‐NF, and GAPDH‐NF (2–5 µL mL^−1^, at the concentration of OD 0.1–0.125) for 16 h. The spheroids were then washed once with PBS and stained with Hoechst 33 342 to stain the nuclei. Cyanine 3 (Cy3), 6‐FAM, and Cy5 filters were used to visualize the signals of KRT14‐NF, FSP1‐NF, and GAPDH‐NF, respectively, on the Olympus FV3000RS inverted confocal microscope. For all samples, Z‐stacks were collected and processed using FIJI software to obtain the sum slices projection images.

### Small Interfering RNA (siRNA) Transfection on 3D Spheroid

To generate spheroids for the use in the NFs sensitivity assay, 2000 NDF, 2000 HaCaT, and 2000 NDF + 2000 HaCaT were seeded per well on day 0. On day1 post‐seeding, the spheroids were transfected with 10 µm of TGFBR1 siRNA duplexes (Thermofisher Scientific) using Lipofectamine 2000 (ThermoFisher Scientific), according to manufacturer's protocol, and incubated at 37 °C for 24 h. The transfection media was then replaced with 100 µL of 1% FBS DMEM with or without TGF*β*1 (10 ng mL^−1^) and incubated at 37° for 24 h. Following these treatments, the spheres were incubated in 100 µL of 1% FBS DMEM containing either KRT14‐NF and GAPDH‐NF (HaCaT spheres) or FSP1‐NF and GAPDH‐NF (NDF spheres) for another 16 h. The spheroids were then washed once with PBS and stained with Hoechst 33 342. Cy3 filter was used to visualize the signals of KRT14‐NF and FSP1‐NF, while GAPDH‐NF signal was detected using Cy5 filter on the Olympus FV3000RS inverted confocal microscope. For all samples, Z‐stacks were collected and processed using FIJI software to obtain the sum slices projection images.

### Monitoring NFs Signal on Normal Mouse Model

Briefly, C57BL/6 was anaesthetized using alfaxalone (≈30–60mg kg^−1^) before generating wounds on the mouse back. Alfaxalone was diluted 1:10 with PBS and injected intramuscularly at 0.1 mL per mouse. The wounds were created using a 4 mm biopsy punch and each mouse has 4 spots. When removing the skin, care was taken to ensure complete removal of the attached connective tissue, and the flap exposure.

To remove variability from individual wounds as a result of anatomical location, the topical applications (GAPDH‐Cy5 NF, PECAM‐Cy3 NF, KRT14‐Cy3 NF, and FSP1‐Cy3 NF) were assigned to 4 wounded spots for experimental group. The order of topical application was rotated clockwise. In the case of the control group, the number of wounded spots was the same for uniformity of the experiment, but only GAPDH NF and GNPs were treated. Moisture cream (Aquaphor) and PBS were mixed at a ratio of 6:4 to make a formulation suitable for treatment of the sample. Then each sample was diluted 1:100 at the final concentration of OD 1 and treated with 10 µL per spot. Overall, topical applications were equally distributed throughout each wounded spot. After applying the sample, mouse was exposed to an infrared heating lamp for 10 min so as to be well absorbed in the wounded spots. NFs mixture was applied on mice every day from day1 to 9. 24 h later, mice were taken for fluorescence imaging with IVIS Spectrum CT (PerkinElmer, Singapore Pt. Ltd.). The field of view, which covered the entire mouse (16 cm), height distance, and optical gain were kept constant throughout the measurement. At the end, the averaged Cy5, Cy3 fluorescence intensity from each region‐of‐interest was recorded.

The wounds were left to heal naturally for 10 days. On the day 2, 4, 7, and 10, 5 mice in the experimental group were euthanized. After euthanasia, the surrounding skin including the wounded spot was removed and stored at −80 ℃ for further study.

### Monitoring NFs Signal on Diabetic Mouse Model

C57BL/6 mice were anaesthetized using isoflurane and became diabetic by intraperitoneal injection of streptozotocin (STZ, Sigma) at 60 mg kg^−1^ in citrate buffer (0.1 m sodium citrate and 0.1 m citric acid, pH 4.5) for 5 consecutive days. The blood glucose levels were measured each day after STZ injection and next 2 continuous weeks by point‐of‐care testing (POCT) Glucose Detection Kit (GlucoCare). Animals with blood glucose above 250 mg dL^−1^ were considered as diabetic ones and were taken for the experimental tests. On the fourth week, the mice were anaesthetized using isoflurane and alfaxalone (≈30–60 mg kg^−1^) before the wound generation. The wounds were created using the same protocol as that in normal mice. The topical application of SNAs and imaging were conducted similarly as that in the normal mice. All animal experiments were approved by the Institutional Animal Care and Use Committees (IACUC) of Korea University, Korea (KOREA‐2021‐0107, KOREA‐2020‐0198).

### Morphological Change of DFU

After Korea University Institutional Review Board approval (IRB No:2021AN0327), DFU treatment was performed based on patient's condition. All experiments were carried out with the full, informed consent of the subjects. Pictures of DFU were taken before and after treatment to demonstrate wound healing stages. NFs were not applied on patients with DFU.

### Statistical Analysis

GraphPad Prism software was used for statistical analysis and graphical representations of data. Statistical tests (*t*‐tests) or one‐way analysis of variance (ANOVA) were carried out to obtain *p* value significance. Unless otherwise stated, data are shown as mean ± standard deviation (SD) or percentage of the mean. To assess statistical significance, analysis of variance (ANOVA) with post‐hoc analysis was performed using a web‐based statistics calculator from http://astatsa.com/OneWay_Anova_with_TukeyHSD/. No statistical method was used to predetermine sample size. All cell‐culture experiments were performed independently at least twice, with multiple duplicate experiments. All in vivo experiments were performed independently with at least 5 subjects. This sample size was sufficient to perform statistical analyses.

## Conflict of Interest

The authors declare no conflict of interest.

## Author Contributions

J.H., C.W., and C.X. designed the project and experiments. J.H., C.W., L.Y., X.N., and C.T.T. performed the experiments and analyzed the data. Y.S., D.J., J.L., J.W.K., Y.K., and J.H. performed the in vivo experiments. J.H., C.W., L.Y., J.W.K., C.T.T., D.H.K., and C.X. wrote the manuscript.

## Supporting information

Supporting information

## Data Availability

The data that support the findings of this study are available on request from the corresponding author. The data are not publicly available due to privacy or ethical restrictions.
