# Supplementary material for: Monitoring Wound Healing with Topically Applied Optical NanoFlare mRNA Nanosensors
Source: Adv Sci (Weinh). 2022 Apr 22;9(18):2104835. doi: 10.1002/advs.202104835 (PMC9218655; doi:10.1002/advs.202104835)
Supplement: Supplementary file 1 — Supporting information [file ADVS-9-2104835-s001.pdf]

ADVANCED SCIENCE
Open Access

# Supporting Information

for *Adv. Sci.*, DOI 10.1002/advs.202104835

Monitoring Wound Healing with Topically Applied Optical NanoFlare mRNA Nanosensors

*Jangsun Hwang, Youngmin Seo, Daun Jeong, Xiaoyu Ning, Christian Wiraja, Lixia Yang, Chew Teng Tan, Jinhyuck Lee, Yesol Kim, Ji Won Kim, Dai Hyun Kim, Jonghoon Choi, Chin Yan Lim, Kanyi Pu, Woo Young Jang* and Chenjie Xu**

Supplementary information for

# Monitoring wound healing with topically applied optical NanoFlare mRNA nanosensors

Jangsun Hwang[1,2], Youngmin Seo[3,4], Daun Jeong[2], Xiaoyu Ning[1,5], Christian Wiraja[1], Lixia Yang[1], Chew Teng Tan[6], Jinhyuck Lee[2], Yesol Kim[7], Ji Won Kim[7], Dai Hyun Kim[8], Jonghoon Choi[7], Chin Yan Lim[6,9], Kanyi Pu[1], Woo Young Jang[2*], Chenjie Xu[10*]

[1] School of Chemical and Biomedical Engineering, Nanyang Technological University, 62 Nanyang Drive, Singapore, 637457

[2] Department of Orthopedic Surgery, College of Medicine, Korea University, 73 Korea-ro, Seongbuk-gu, Seoul, Republic of Korea, 02841

[3] School of Electrical and Electronic Engineering, Yonsei University, 50 Yonsei-ro, Seodaemun-gu, Seoul, Republic of Korea, 03722

[4] Department of Research & Development, OID Ltd, 249-2, 123 Osongsaengmyeong-ro, Osong-eup, Heungdeok-gu, Cheongju-si, Chungcheongbuk-do, Republic of Korea, 28160

[5] NTU Institute for Health Technologies, Interdisciplinary Graduate School, Nanyang Technological University, 61 Nanyang Drive, Singapore, 637335

[6] A*STAR Skin Research Labs, Agency for Science, Technology and Research, 8A Biomedical Grove, Singapore 138648

[7] School of Integrative Engineering, Chung-Ang University, 84, Heukseok-ro, Dongjak-gu, Seoul, Republic of Korea, 06974

[8] Department of Dermatology, College of Medicine, Korea University, 73 Korea-ro, Seongbuk-gu , Seoul, Republic of Korea, 02841

[9] Department of Biochemistry, Yong Loo Lin School of Medicine, National University of Singapore, MD 7, 8 Medical Drive, Singapore 117596

[10] Department of Biomedical Engineering, City University of Hong Kong, 83 Tat Chee Avenue, Kowloon, Hong Kong SAR, China

[*] Correspondence to chenjie.xu@cityu.edu.hk and opmanse@korea.ac.kr

| Flare | −ΔG(Duplex) (kcal/mol)* | |
|---|---|---|
| | FSP1 (human) | FSP1 (mouse) |
| 12 nt | 13.7 | |
| 14 nt | 16.9 | |
| 15 nt | 18.7 | 21.4 |
| 16 nt | 21.0 | |
| 18 nt | 24.5 | |
| Target | 53.4 | 58.6 |
| | PECAM1 (human) | PECAM1 (mouse) |
| 15 nt | 19.4 | 21.4 |
| Target | 48.9 | 61.4 |
| | KRT14 (human) | KRT14 (mouse) |
| 15 nt | 20.8 | 20.8 |
| Target | 56.8 | 56.8 |
| | GAPDH (human) | GAPDH (mouse) |
| 15 nt | 19.6 | 20.9 |
| Target | 57.8 | 59.9 |

*Predicted using RNA Structure

**Table S1. The predicted free energy change of the R-F (recognition-flare) duplex with varied flare length of 12, 14, 15, 16, and 18 nt (human and mouse).**

| Binding free energy of Nanoflare | R+T | R+F | RF/FT (%) | GC contents (%) | Recovery kinetics , Saturation |
|---|---|---|---|---|---|
| | −ΔG(Duplex) (kcal/mol) | | | | |
| FSP1 | 53.4 | 18.7 | 35 | 46 | (high, fast) |
| KRT14 | 56.8 | 20.8 | 36.6 | 53 | (low, slow) |
| PECAM1 | 48.9 | 19.4 | 39.7 | 46 | (low, fast) |
| GAPDH | 57.8 | 19.6 | 33.9 | 53 | (high, slow) |

**Table S2. Recovery kinetics and saturation time of NFs**

| Protein | Gene | Forward | Reverse |
|---|---|---|---|
| FSP-1 | S100A4 | 5′-TGAGCAACTTGGACAGCAACA-3′ | 5′-ATTTCTTCCTGGGCTGCTTATCT-3′ |
| CTGF | CCN2 | CAGCATGGACGTTCGTCTG | AACCACGGTTTGGTCCTTGG |
| Collagen-1 | COL1A1 | TCTGCGACAACGGCAAGGTG | GACGCCGGTGGTTTCTTGGT |
| HSP47 | SERPINH1 | AACCGTGGCTTCATGGTGACTC | TGATGAGGCTGGAGAGCTTGTG |
| α-SMA | ACTA2 | CTATGCCTCTGGACGCACAACT | CAGATCCAGACGCATGATGGCA |
| CD24 | CD24 | CACGCAGATTTATTCCAGTGAAAC | GACCACGAAGAGACTGGCTGTT |
| CD26 | DPP4 | AAAGGCACCTGGGAAGTCATCG | CAGCTCACAACTGAGGCATGTC |
| CD90 | THY1 | GAAGGTCCTCTACTTATCCGCC | TGATGCCCTCACACTTGACCAG |
| SCA-1 | ATXN1 | AGATCGACTCCAGCACCGTAGA | CTCTACCAAAACTTCAACGCTGAC |
| DDR2 | DDR2 | AACGAGAGTGCCACCAATGGCT | ACTCACTGGCTTCAGAGCGGAA |
| MMP-2 | MMP2 | AGCGAGTGGATGCCGCCTTTAA | CATTCCAGGCATCTGCGATGAG |
| Vimentin | VIM | AGGCAAAGCAGGAGTCCACTGA | ATCTGGCGTTCCAGGGACTCAT |
| **Protein** | **Gene** | **Forward** | **Reverse** |
| Keratin 10 | KRT10 | 5′-CCTGCTTCAGATCGACAATGCC-3′ | 5′-ATCTCCAGGTCAGCCTTGGTCA-3′ |
| Keratin 14 | KRT14 | TGCCGAGGAATGGTTCTTCACC | GCAGCTCAATCTCCAGGTTCTG |
| Keratin 1 | KRT1 | CAGCATCATTGCTGAGGTCAAGG | CATGTCTGCCAGCAGTGATCTG |
| Keratin 5 | KRT5 | GCTGCCTACATGAACAAGGTGG | ATGGAGAGGACCACTGAGGTGT |
| Keratin 16 | KRT16 | CTACCTGAGGAAGAACCACGAG | CTCGTACTGGTCACGCATCTCA |
| Involucrin | IVL | GGTCCAAGACATTCAACCAGCC | TCTGGACACTGCGGGTGGTTAT |
| Filaggrin | FLG | GCTGAAGGAACTTCTGGAAAAGG | GTTGTGGTCTATATCCAAGTGATC |
| Transglutaminase | TGM1 | GAACGACTGCTGGATGAAGAGG | CTTGATGGACTCCACAGAGCAG |
| P2X purinoceptor 7 | P2RX7 | CGACTAGGAGACATCTTCCGAG | GCAGTGATGGAACCAACGGTCT |
| P2Y purinoceptor 2 | P2RY2 | CGAGGACTTCAAGTACGTGCTG | GTGGACGCATTCCAGGTCTTGA |
| Caspase 14 | CASP14 | GGTGGATGTGTTCACGAAGAGG | CCTTCTTGAACCAGCTCTGCTTC |
| SPRR2A | SPRR2A | CAGTGCCAGCAGAAATATCCT | CCAAATATCCTTATCCTTTCTTGG |
| **Protein** | **Gene** | **Forward** | **Reverse** |
| PECAM-1/CD31 | PECAM1 | 5′-AAGTGGAGTCCAGCCGCATATC-3′ | 5′-ATGGAGCAGGACAGGTTCAGTC-3′ |
| KDR/VEGFR2 | KDR | GGAACCTCACTATCCGCAGAGT | CCAAGTTCGTCTTTTCCTGGGC |
| VEGFR1 | FLT1 | CCTGCAAGATTCAGGCACCTATG | TTGCAGTGCTCACCTCTGATT |
| CD34 | CD34 | CCTCAGTGTCTACTGCTGGTCT | GGAATAGCTCTGGTGGCTTGCA |
| Endoglin/CD105 | ENG | CGGTGGTCAATATCCTGTCGAG | AGGAAGTGTGGGCTGAGGTAGA |
| MCAM/CD146 | MCAM | ATCGCTGCTGAGTGAACCACAG | CTACTCTCTGCCTCACAGGTCA |
| ICAM-1 | ICAM1 | AGCGGCTGACGTGTGCAGTAAT | TCTGAGACCTCTGGCTTCGTCA |
| VCAM-1 | VCAM1 | GATTCTGTGCCCACAGTAAGGC | TGGTCACAGAGCCACCTTCTTG |
| VE-Cadherin /CD144 | CDH5 | GAAGCCTCTGATTGGCACAGTG | TTTTGTGACTCGGAAGAACTGGC |
| vWF | VWF | CCTTGAATCCCAGTGACCCTGA | GGTTCCGAGATGTCCTCCACAT |
| Tie-2 | TEK | GGTCAAGCAACCCAGCCTTTTC | CAGGTCATTCCAGCAGAGCCAA |
| EDG1/S1PR1 | S1PR1 | CCTGTGACATCCTCTTCAGAGC | CACTTGCAGCAGGACATGATCC |

**Table S3. Primer information.**

| Gene | Nucleotide | | Sequence |
|---|---|---|---|
| **FSP1** | Human Recognition | | 5′- ATTTCTTCCTGGGCTGCTTATCTGGGAAAAAAAAA-SH 3′ |
| | Mouse Recognition | | 5′- ACTTCTTCCGGGGCTCCTTATCTGGGCAAAAAAA-SH 3 |
| | Flare | | |
| | Length | Human 18 nt | 3′- CCCGACGAATAGACCCTT- **FAM** -5′ |
| | | Human 16 nt | 3′- CGACGAATAGACCCTT- **FAM** -5′ |
| | | Human 15 nt | 3′- GACGAATAGACCCTT- **FAM** -5′ |
| | | Mouse 15 nt | 3′- GAGGAATAGACCCGT- **Cy3** -5′ |
| | | Human 14 nt | 3′- ACGAATAGACCCTT- **FAM** -5′ |
| | | Human 12nt | 3′- GAATAGACCCTT- **FAM** -5′ |
| **PECAM1** | Human Recognition | | 5’-ATGGAGCAGGACAGGTTCAGTCTTTCAC AAAAAA-SH-3’ |
| | Mouse Recognition | | 5’-TTAAGGGAGCCTTCCGTTCTTAGGGTCGACAAAAAA-SH-3’ |
| | Human Flare | | 3’-CCAAGTCAGAAAGTG-**Cy3**-5’ |
| | Mouse Flare | | 3’-CAAGAATCCCAGCTG-**Cy3**-5’ |
| **KRT14** | Human Recognition | | 5′- GACTGCAGCTCAATCTCCAGGTTCTGCAAAAAAA-SH -3′ |
| | Same as human | | |
| | Human Flare | | 3′- AGAGGTCCAAGACGT- **Cy3** -5′ |
| | Same as human | | |
| **GAPDH** | Human Recognition | | 5′- GATGGCATGGACTGTGGTCATGAGTCCTAAAAAA-3′ |
| | Mouse Recognition | | 5′- GATGGCATGGACTGTGGTCATGAGCCCTAAAAAA-3′ |
| | Human Flare | | 3’-CACCAGTACTCAGGA-**Cy5**-5’ |
| | Mouse Flare | | 3’-CACCAGTACTCGGGA-**Cy5**-5’ |

**Table S4. Sequence of recognitions and flares for NanoFlare.**

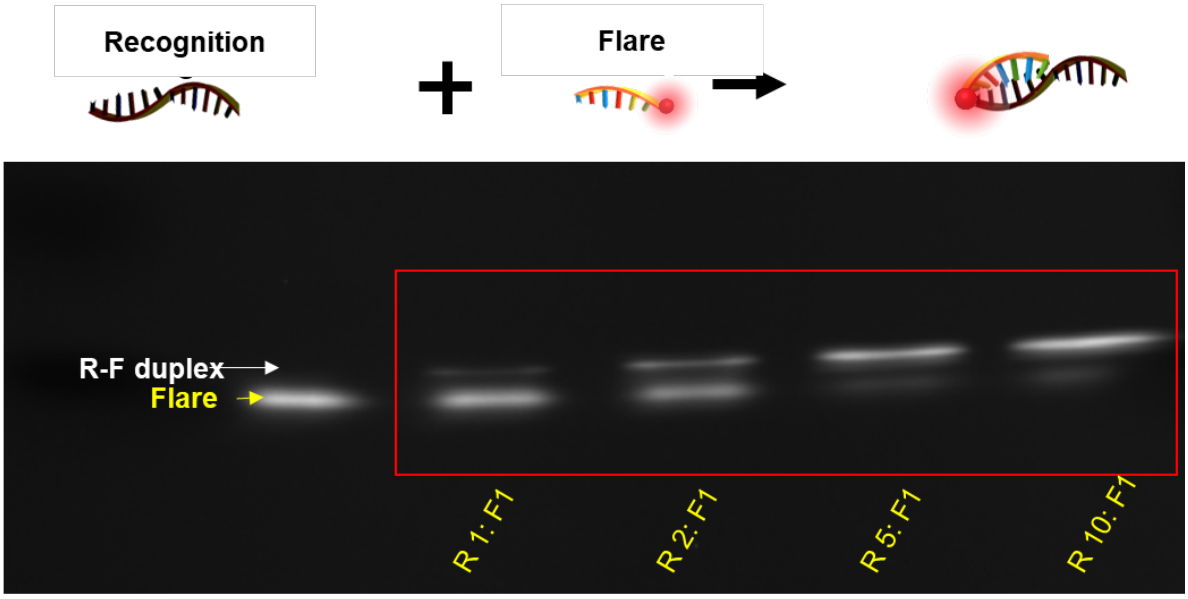


**Supplementary Figure S1. Optimization of R:F ratio for the R-F duplex hybridization.** Polyacrylamide gel electrophoresis of R-F hybridization under different R:F ratios (R and F concentration: 100 µM, R: F=v/v).

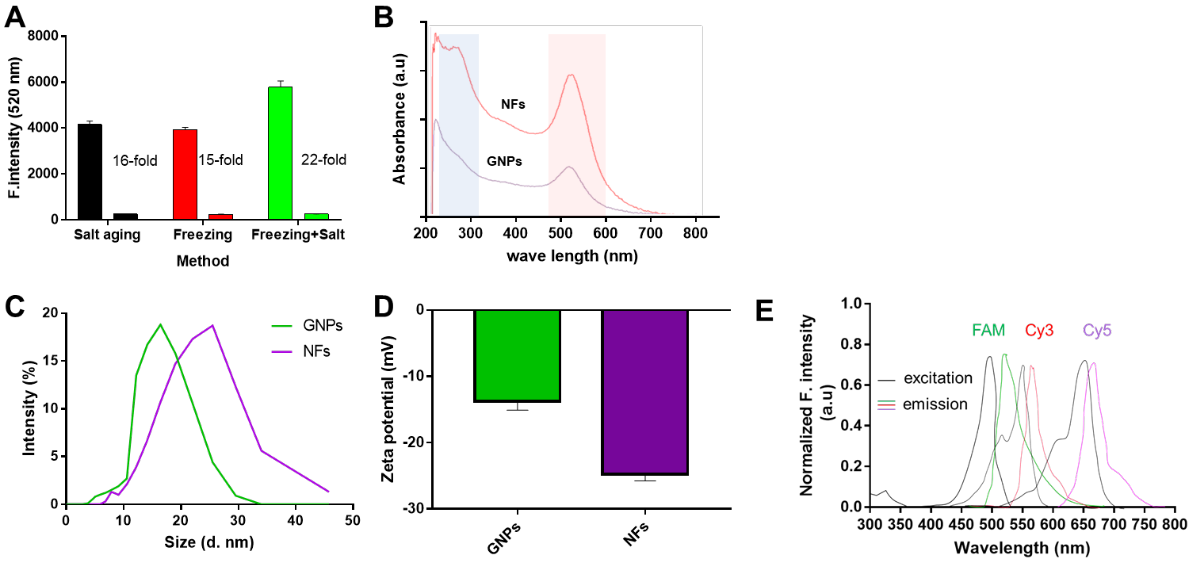


**Supplementary Figure S2. Characterization of NanoFlares (NFs): (A)** The change of fluorescence signal upon the mixing of target sequence and NFs made from different methods; **(B)** Absorbance spectra of gold nanoparticles (GNPs) and NF; **(C)** Hydrodynamic diameters; **(D)** Zeta-potentials of GNPs and NFs (n=3, values are means ± s.d.); **(E)** Excitation and emission spectra of FAM (493-517 nm), Cy3 (555-569 nm), and Cy5 (651-670 nm).

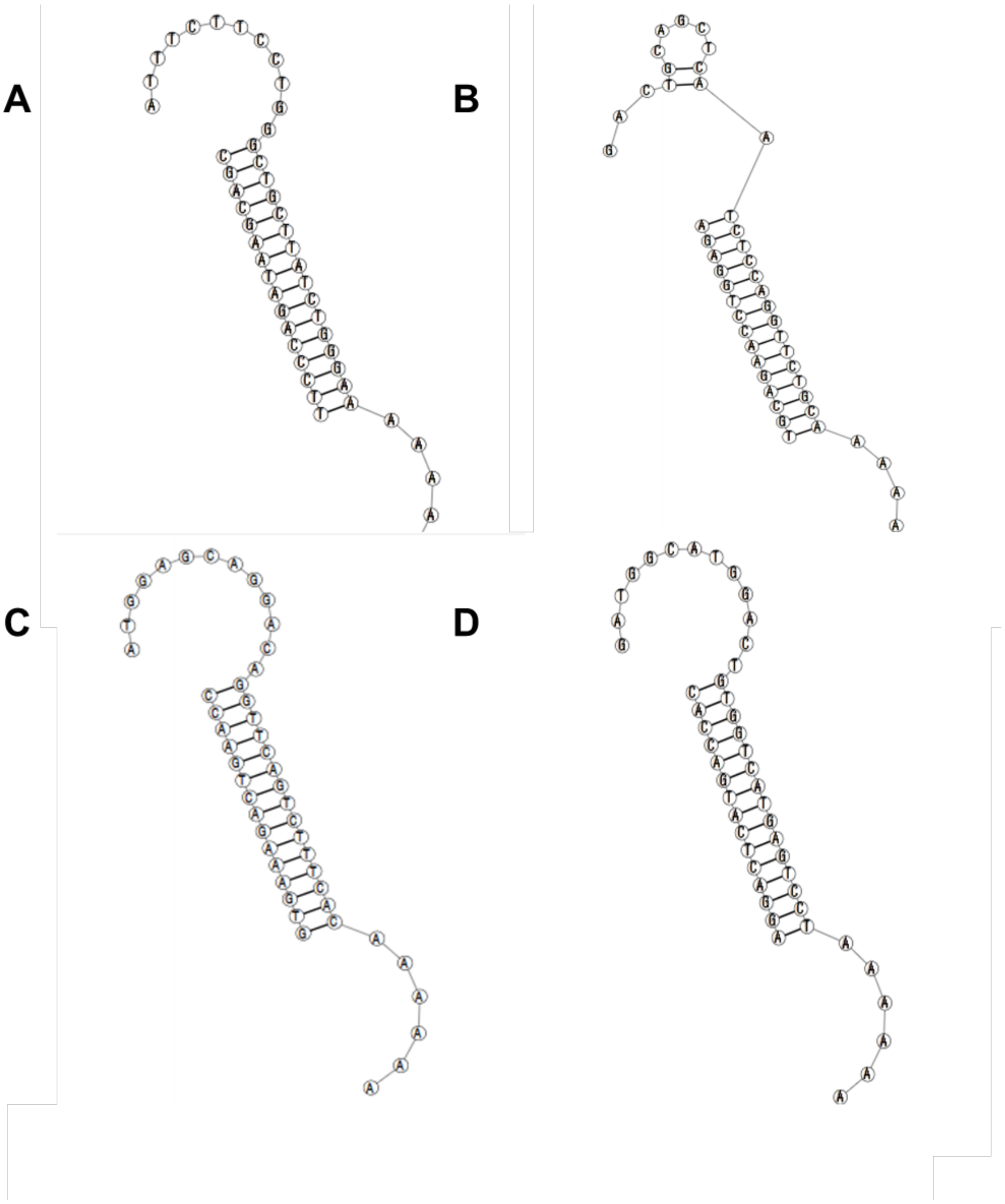


**Supplementary Figure S3. R+F selection: (A)** Possible secondary structure prediction of the R-F (recognition-flare) duplex for FSP1-NFs; **(B)** KRT14-NFs; **(C)** PECAM1-NFs; **(D)** GAPDH-NFs.

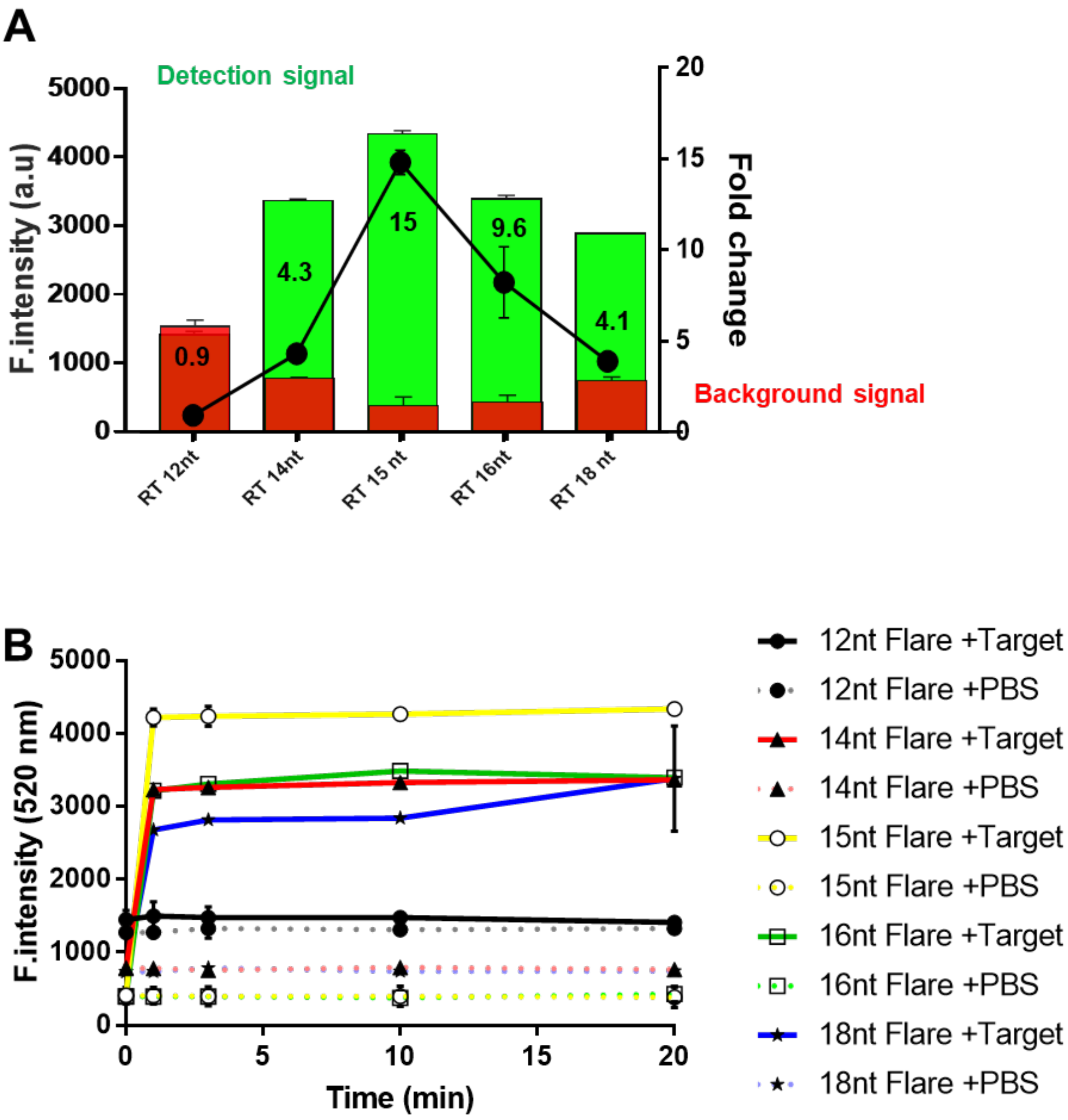


**Supplementary Figure S4. Upon the addition of target sequence, the changes of fluorescence signals of FSP1-NFs that were synthesized with 12, 14, 15, 16, 18 and 20 nucleotide-long flare sequences: (A)** quantification of the fluorescence signal changes; **(B)** the change of fluorescence over time (n=3, NFs concentration: O.D 0.4, Target= 2 µM, values are means ± s.d.).

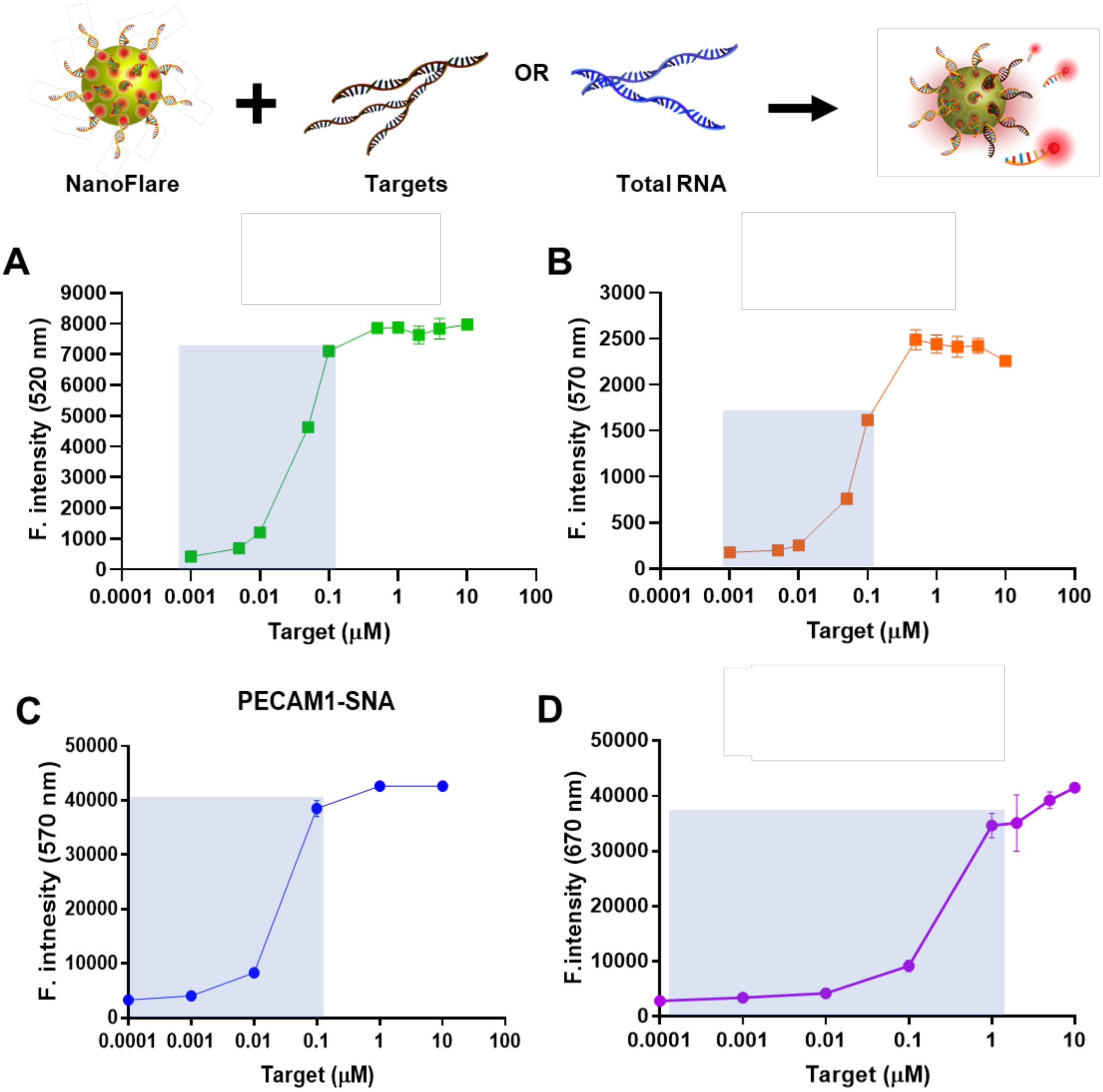


**Supplementary Figure S5.** Restoration of (**A**) FSP1-NFs; (**B**) KRT14-NFs; (**C**) PECAM1-NFs; (**D**) GAPDH-NFs fluorescence under different concentrations of target sequences (n=3, values are means ± s.d.).

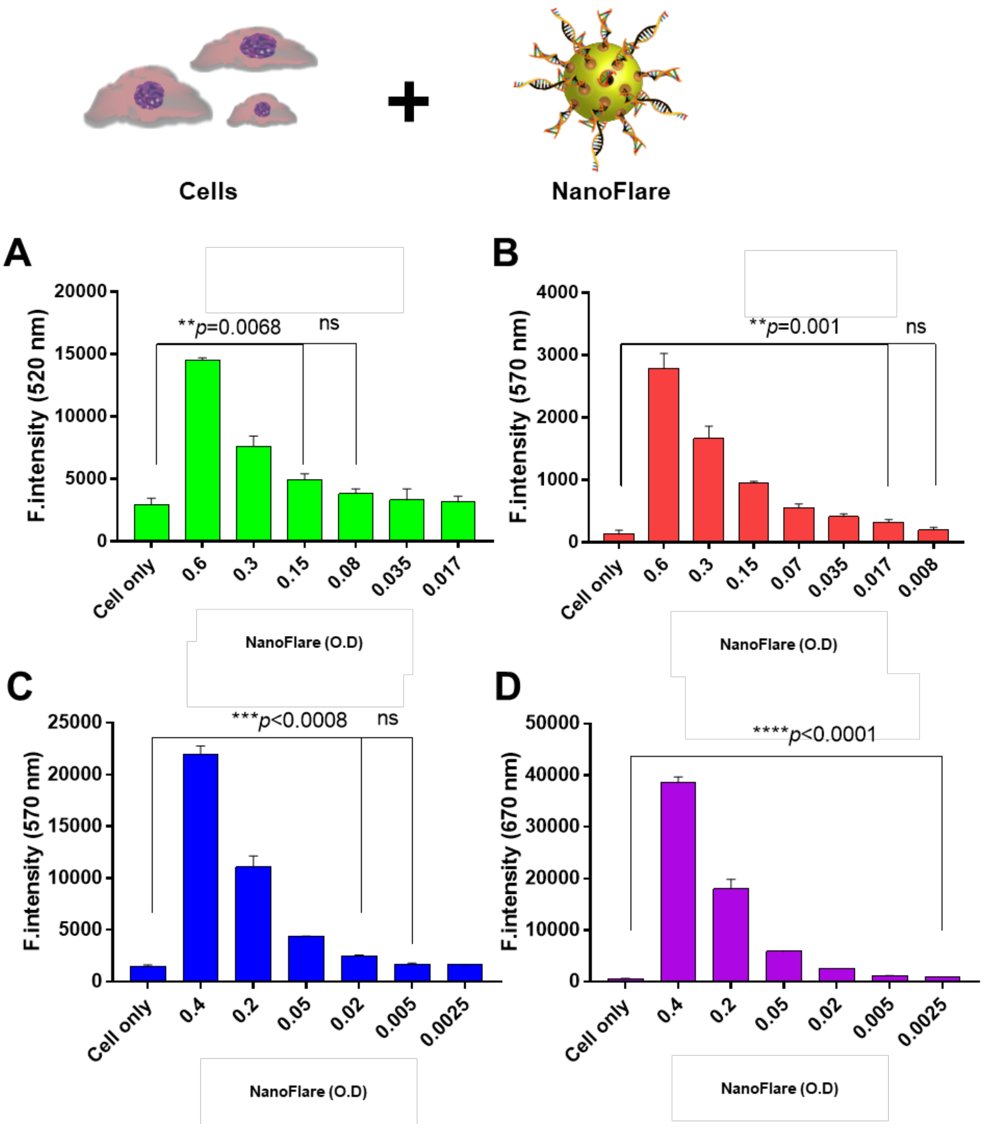


**Supplementary Figure S6.** Optimization of **(A)** FSP1-NFs; **(B)** KRT14-NFs; **(C)** PECAM1-NFs; **(D)** GAPDH-NFs working concentrations using cell experiments. (n=3, 5x10$^4$/mL cells were labeled with NFs in 24 hrs, (***p*<0.01, *** *p* <0.001, **** *p* <0.0001, and ns= not significant, n=3, values are means ± s.d.).

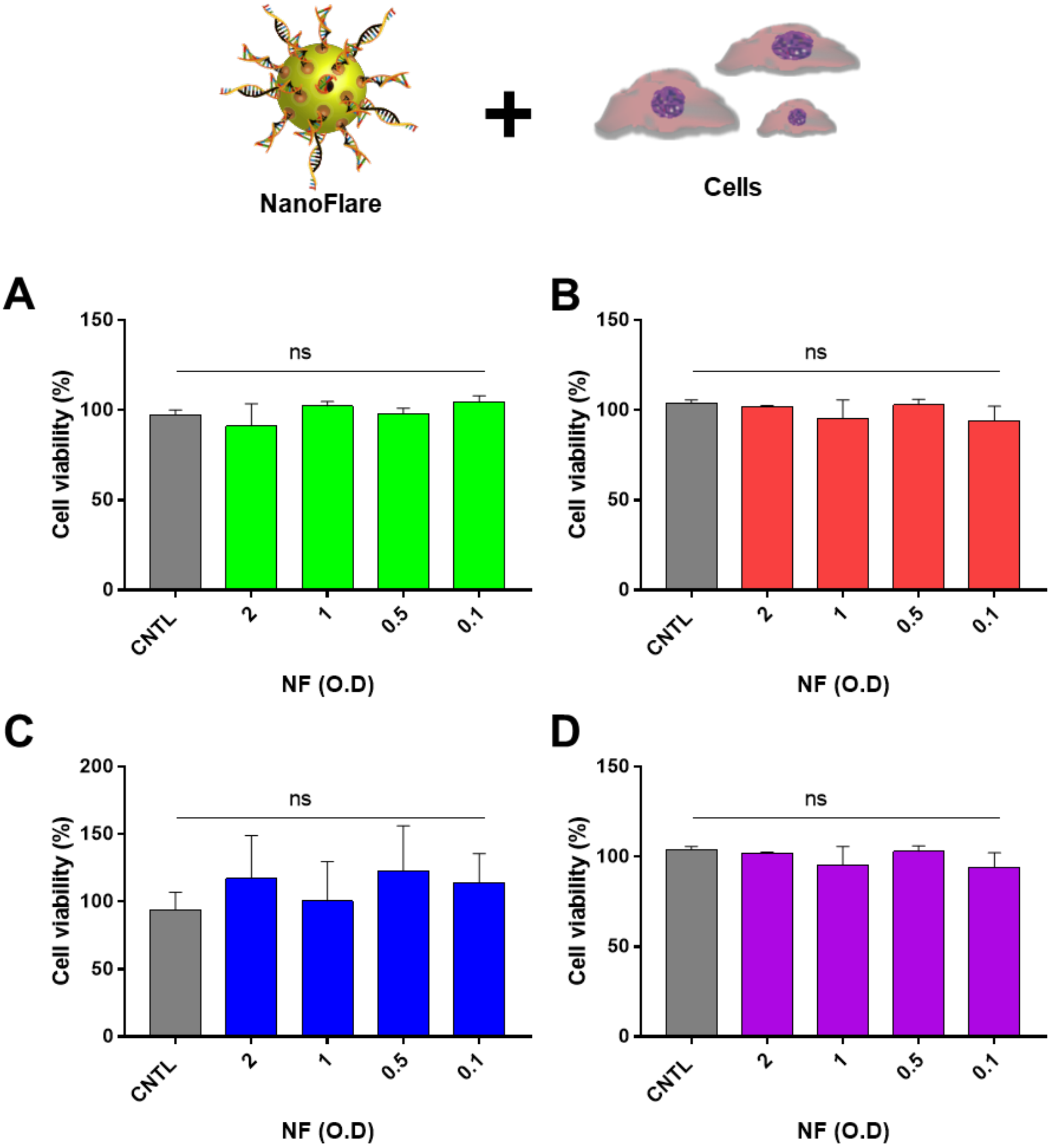


**Supplementary Figure S7.** Cytotoxicity study of **(A)** FSP1-NFs on NDF; **(B)** KRT14-NFs on HaCaT; **(C)** PECAM1- NFs on HUVEC; **(D)** GAPDH-NFs on HUVEC (CNTL=no treat, ns= not significant, $5x10^4$/mL cells were cultured with NFs in 24 hrs, n=3, values are means ± s.d.).

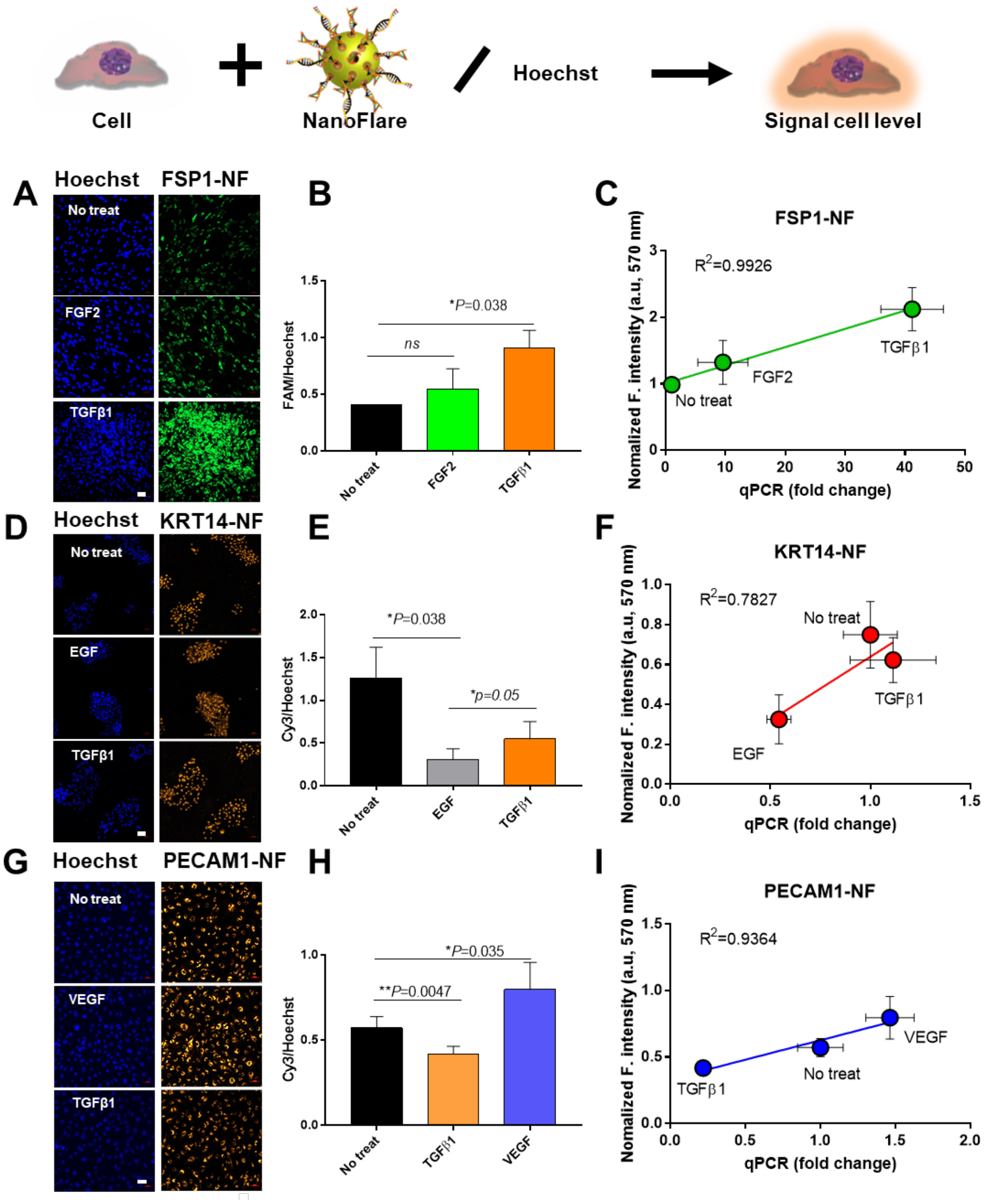


**Supplementary. Figure S8.** Evaluation of target gene expression in cells by NFs.(A) Fluorescence images of FSP1-NF labeled NDFs (Control: no treat, FGF2: 40 ng/mL, TGFβ1: 40 ng/mL, scale bar =50 µm); (B) Normalized FSP1-NF signal by taking Hoechst as the reference; (C) Correlation between the normalized FSP1-NF signal and qPCR results; (D) Fluorescence images of KRT14-NF labeled HaCat (Control: no treat, EGF: 40 ng/mL, TGFβ1: 40 ng/mL, scale bar =50 µm); (E) Normalized KRT14-NF signal by taking Hoechst as the reference; (F) Correlation between the normalized KRT14-NF signal and qPCR results; (G) Fluorescence images of PECAM1-NF labeled HUVEC (Control: no treat, VEGF: 40 ng/mL, TGFβ1: 40 ng/mL, scale bar =50 µm); (H) Normalized PECAM1-NF signal by taking Hoechst as the reference; (I) Correlation between the normalized PECAM1-NF signal and qPCR results (NFs=O.D 0.1 for FSP-NF, 0.0125 for KRT14-NF and PECAM1-NF, cells were starved and cultured in serum reduced media for 2 days before NF treatment, $**p<0.01$, $***\ p<0.001$, and ns= not significant, n=3, values are means ± s.d.).

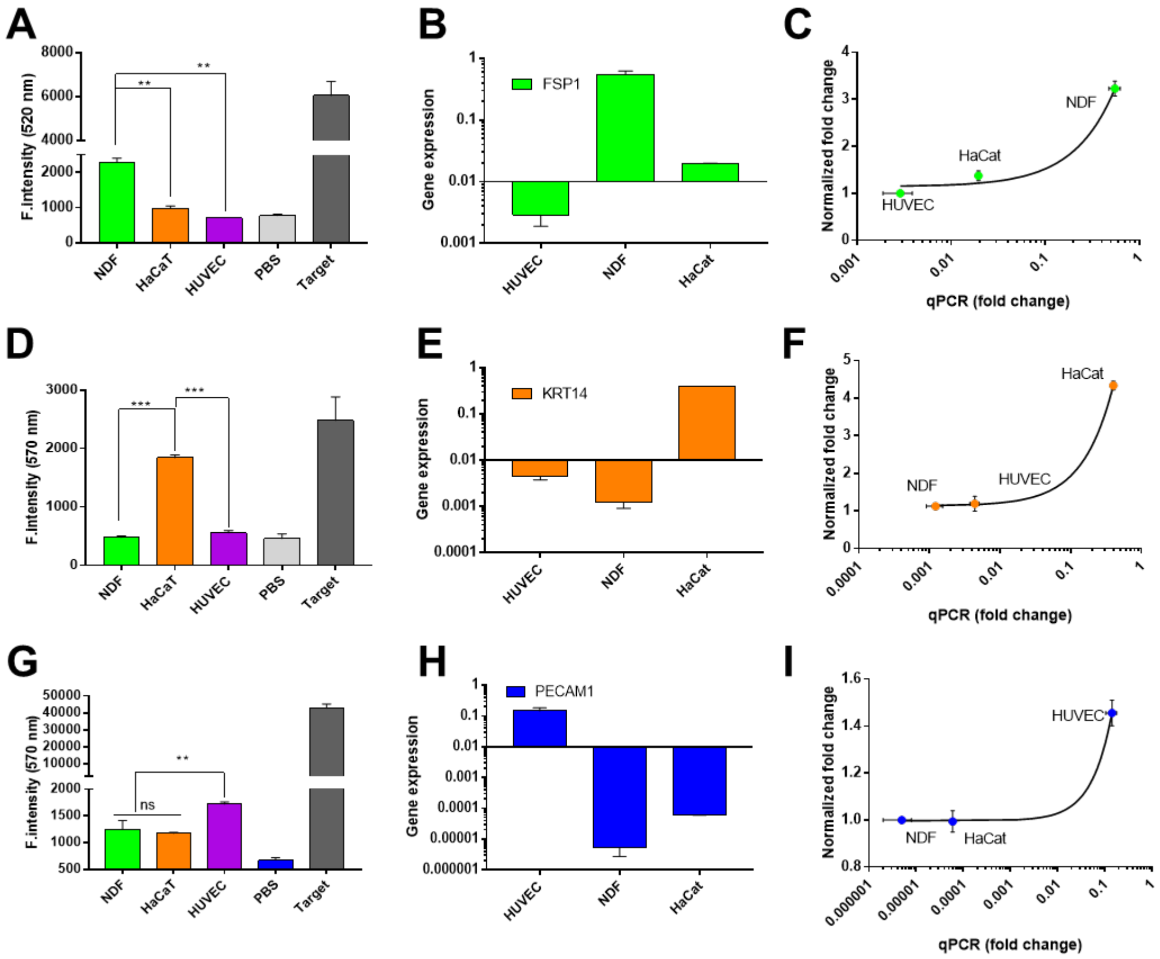


**Supplementary Figure S9**. **Quantification of target gene expression in cells: (A)** FSP1 expression in three types of cells evaluated by FSP1-NF (total RNA of 100 ng/µL, NF=O.D 0.4); **(B)** qPCR evaluation of FSP1 expression in cells; **(C)** Correlation between the normalized FSP1-NF signal and qPCR results; **(D)** KRT14 expression in three types of cells evaluated by KRT14-NF (total RNA of 100 ng/µL, NF=O.D 0.4); **(E)** qPCR evaluation of KRT14 expression in cells; **(F)** Correlation between the normalized KRT14-NF signal and qPCR results; **(G)** PECAM1 expression in three types of cells evaluated by PECAM1-NF (total RNA of 100 ng/µL, NF=O.D 0.4); **(H)** qPCR evaluation of PECAM1expression in cells; **(I)** Correlation between the normalized PECAM1-NF signal and qPCR results. Values are means ± s.d. (**$p$<0.01, *** $p$ <0.001, and ns= not significant, n=3, values are means ± s.d.).

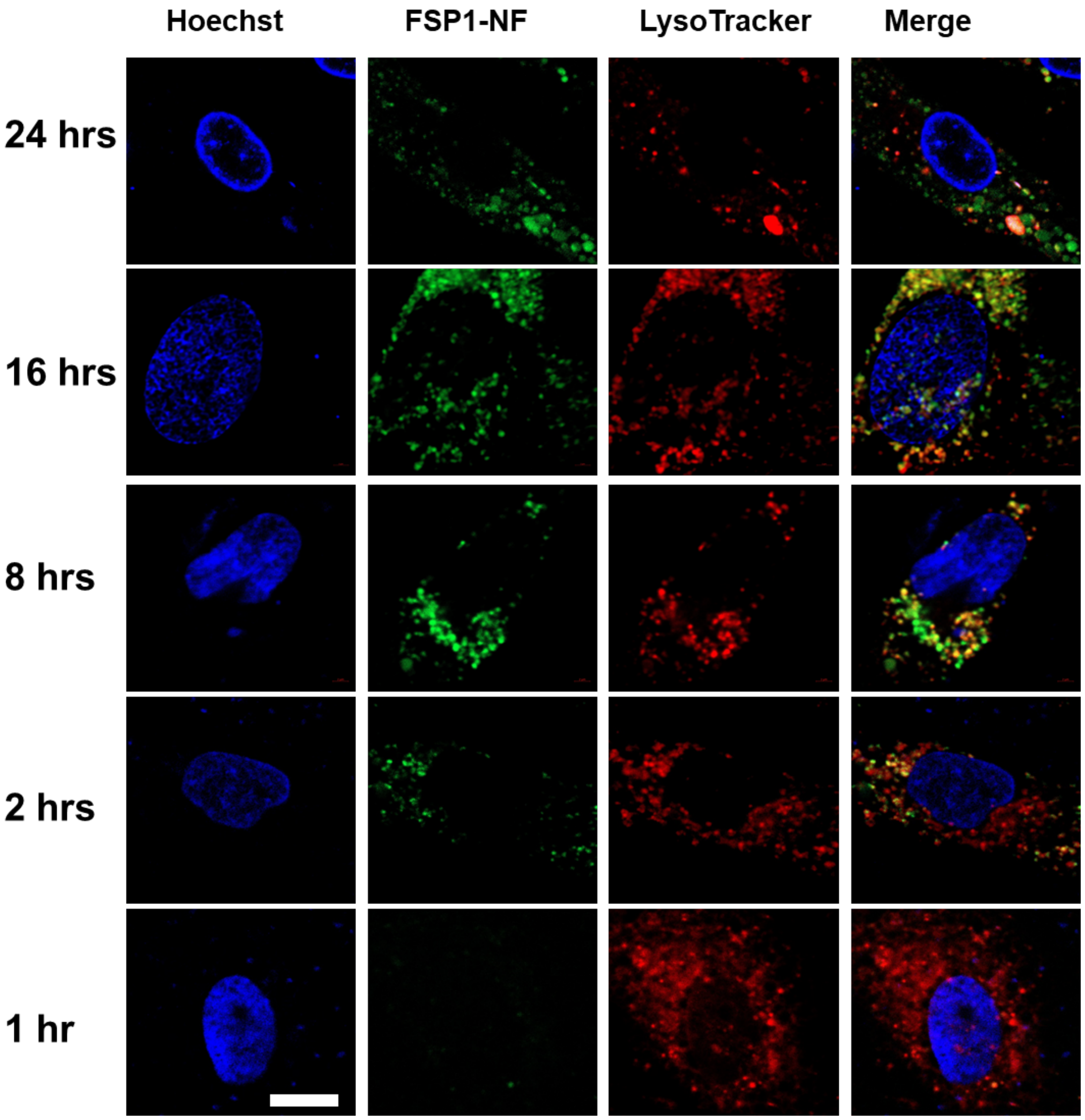


**Supplementary Figure S10. Confocal fluorescence images of NDFs labeled with FSP1-NFs at different time points.** Cells were pre-stained with Lysotracker. Scale bar is 20 µm, Cy3: lysotracker, Blue: Hoechst, and Green: FSP1-NFs.

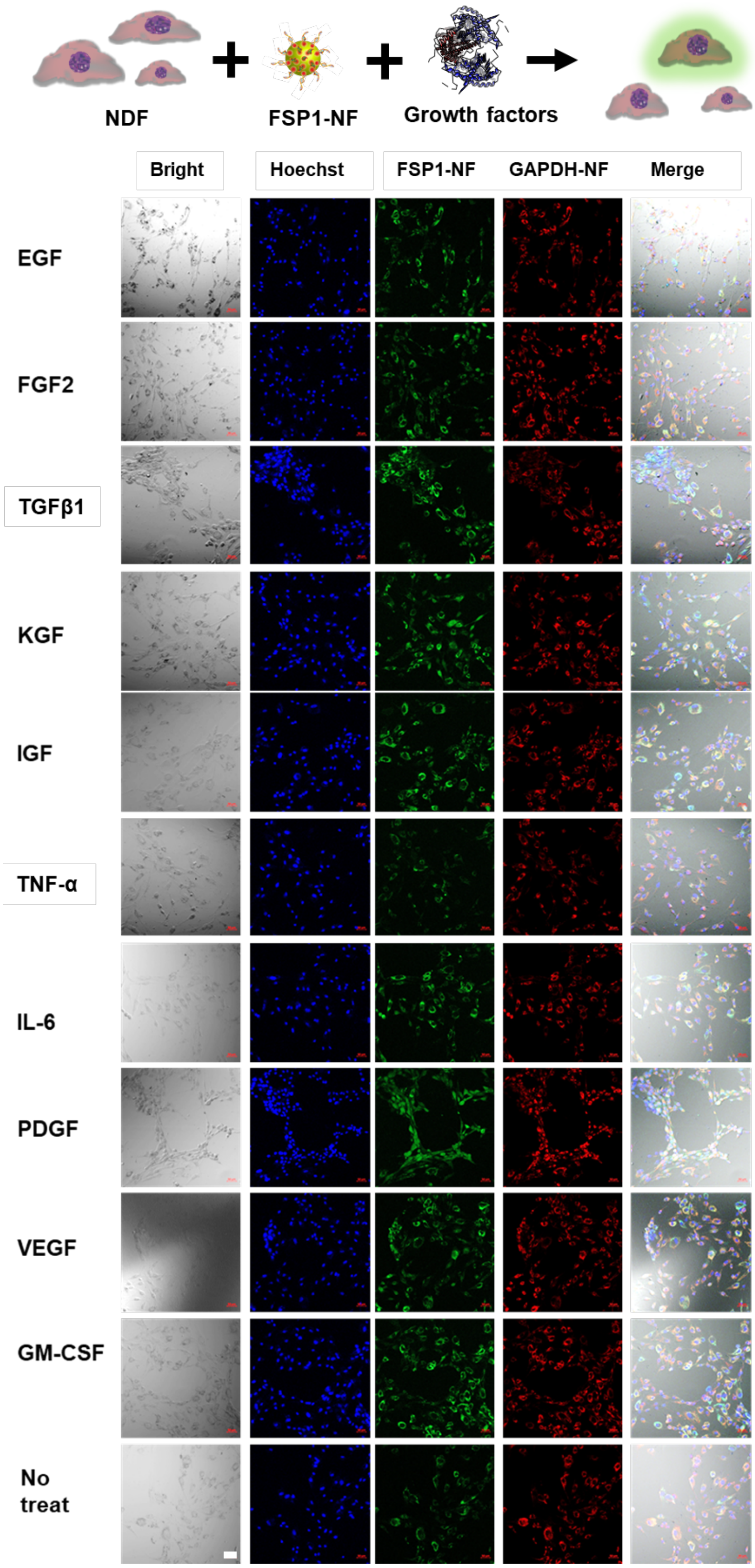


**Supplementary Figure S11. Fluorescence images of NDF treated with 10 growth factors and NFs.** (CNTL: no treat, EGF: 40 ng/mL, FGF2: 40 ng/mL, TGFβ1: 40 ng/mL, KGF: 40 ng/mL, IGF:40 ng/mL, TNF-α: 40 ng/mL, IL-6: 40 ng/mL, PDGF: 8 ng/mL, VEGF: 20 ng/mL, GM-CSF: 20 ng/mL) Scale bar is 50 μm. The concentrations of FSP1-NF and GAPDH-NF were O.D 0.4 and 0.0125 respectively.

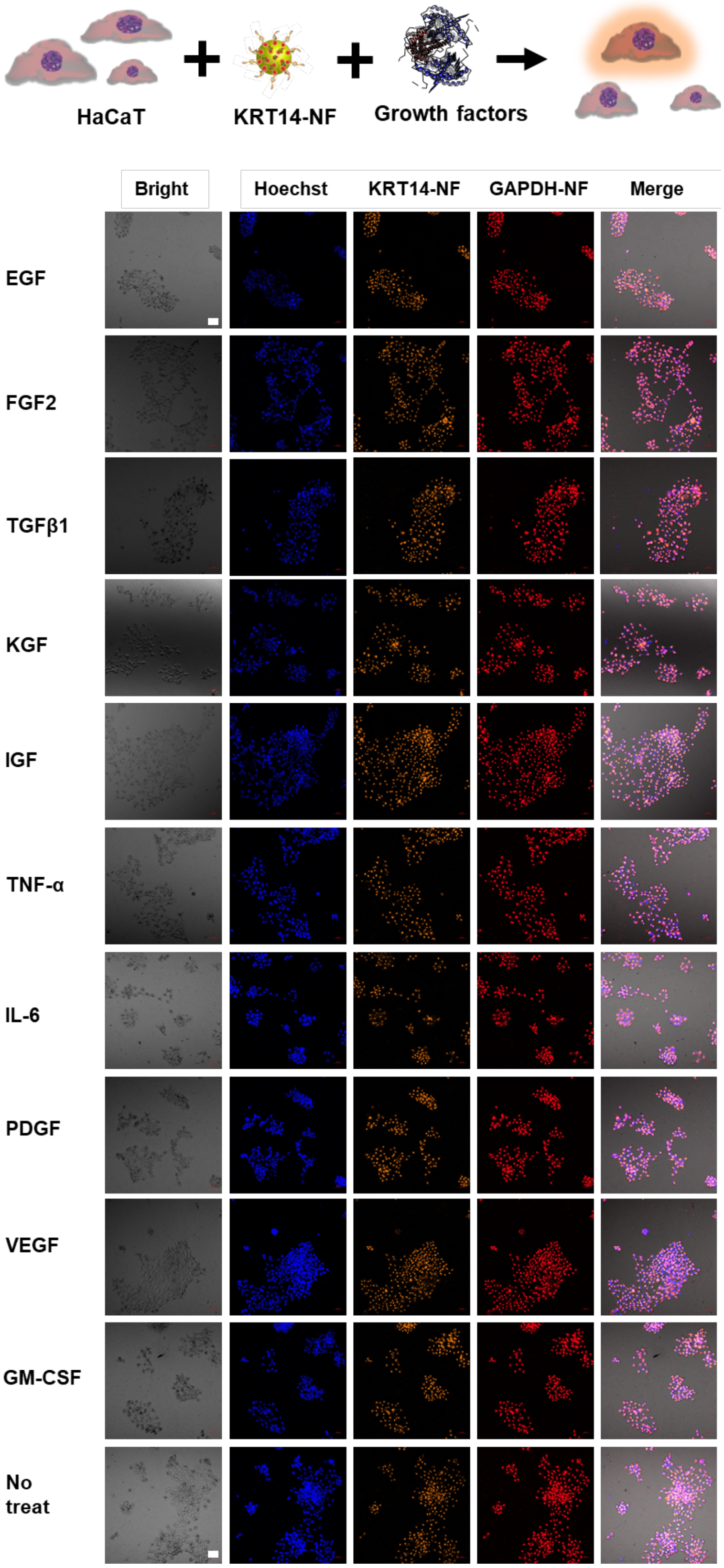


**Supplementary Figure S12. Fluorescence images of HaCaT treated with 10 growth factors and NFs.** (CNTL: no treat, EGF: 40 ng/mL, FGF2: 40 ng/mL, TGFβ1: 40 ng/mL, KGF: 40 ng/mL, IGF:40 ng/mL, TNF-α: 40 ng/mL, IL-6: 40 ng/mL, PDGF: 8 ng/mL, VEGF: 20 ng/mL, GM-CSF: 20 ng/mL, scale bar =50 μm, Both concentrations of KRT14-NF and GAPDH-NF were 0.0125.

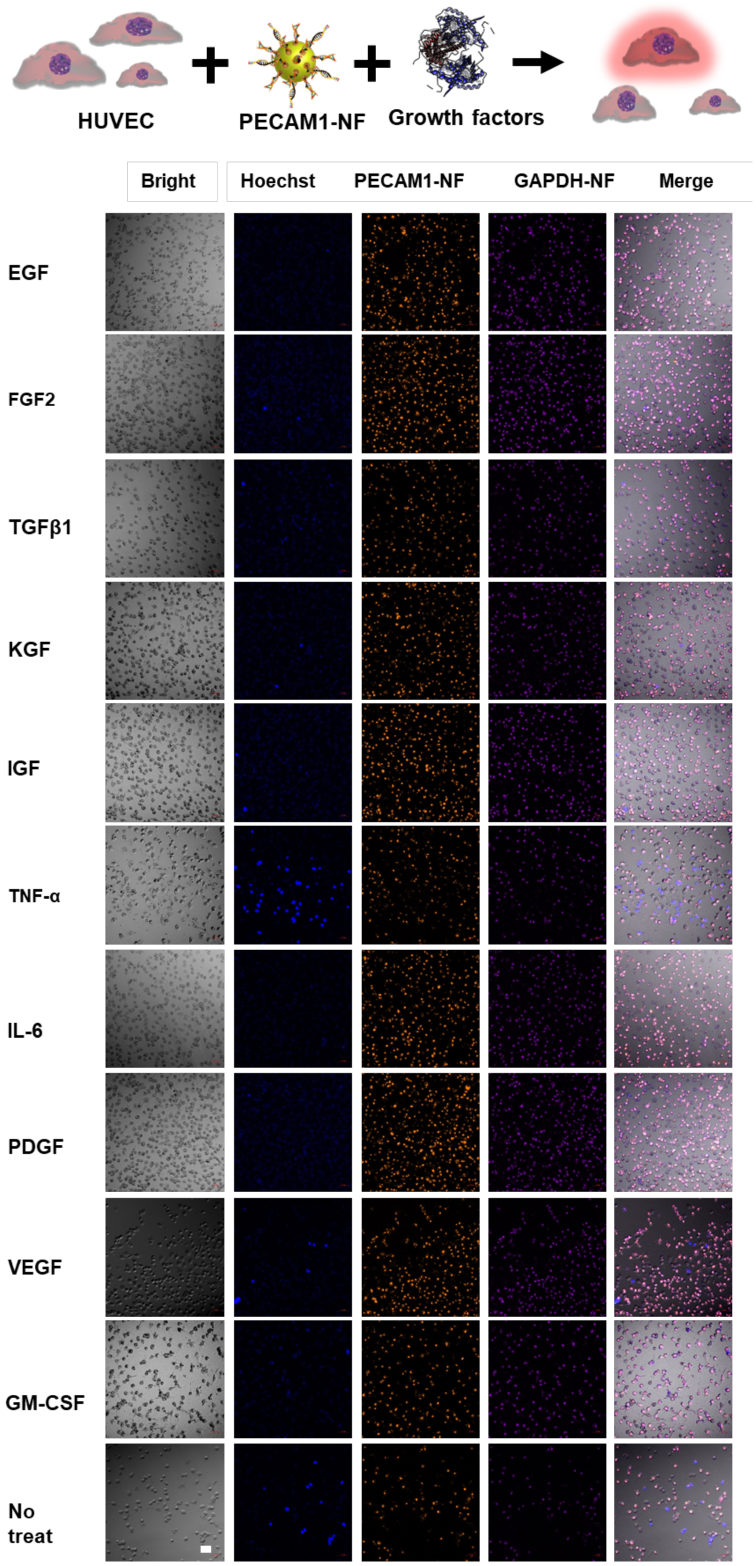


**Supplementary Figure S13. Fluorescence images of HUVEC treated with 10 growth factors and NFs.** (CNTL: no treat, EGF: 40 ng/mL, FGF2: 40 ng/mL, TGFβ1: 40 ng/mL, KGF: 40 ng/mL, IGF:40 ng/mL, TNF-α: 40 ng/mL, IL-6: 40 ng/mL, PDGF: 8 ng/mL, VEGF: 20 ng/mL, GM-CSF: 20 ng/mL, scale bar =50 μm, Both concentrations of PECAM1-NF and GAPDH-NF were O.D 0.0125.

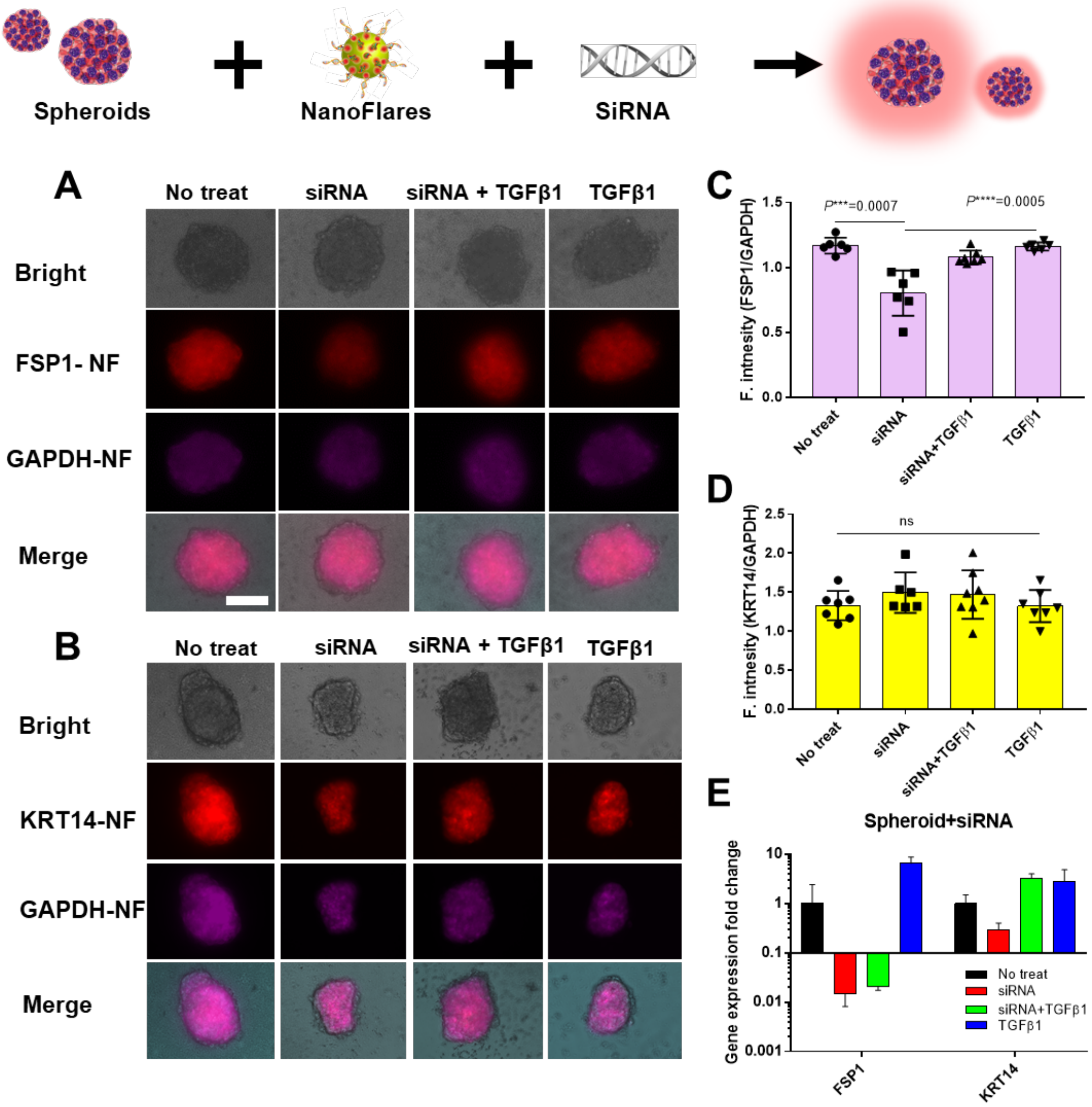


**Supplementary Figure S14. Monitoring the cellular gene changes in 3D spheroid using NFs: (A)** Fluorescence images of FSP1-NF labeled NDF spheroids that were pre-treated with TGFBR1 siRNA, TGFBR1 siRNAs +TGFβ1, and TGFβ1. (n=10, scale bar =100 μm); **(B)** Fluorescence images of KRT14-NF labeled HaCaT spheroids that were pre-treated with TGFBR1 siRNA, TGFBR1 siRNAs +TGFβ1, and TGFβ1 (n=10, scale bar =100 μm); **(C)** Quantification of FSP1 NF signals in **A**; **(D)** Quantification of KRT14 NF signals in B; **(E)** qPCR results of FSP1 and KRT14 expression in 3D spheroids. Values are means ± s.d. (*** $p$ <0.001, **** $p$ <0.0001, and ns= not significant, values are means ± s.d.).

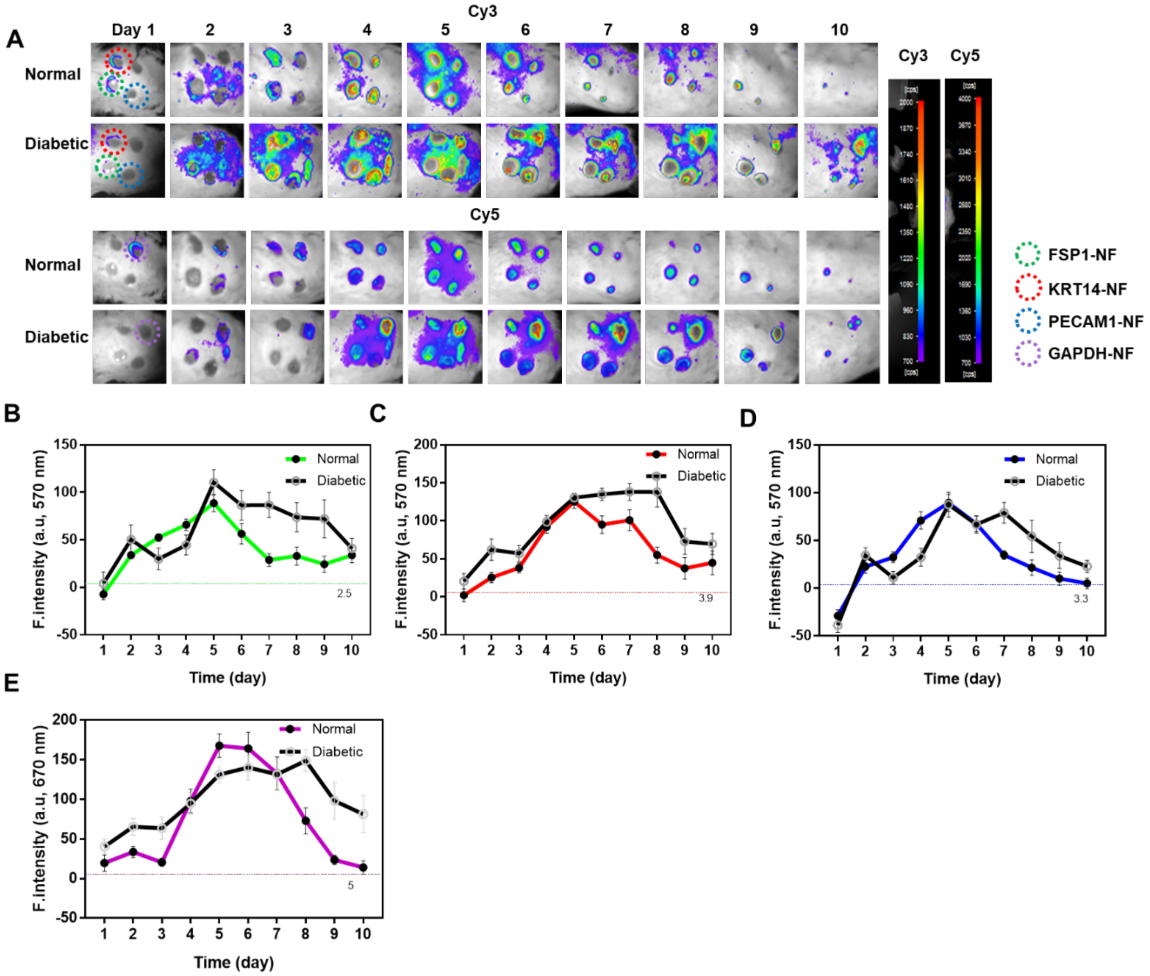


**Supplementary Figure S15. Tracking wound healing process using NFs on normal and diabetic wound healing models:** **(A)** IVIS imaging of mice post NFs application (FSP1-NF treated wound: green circles; KRT14-NF treated wound: red circle; PECAM1-NF treated wound: blue circles; GAPDH-NF treated wound: purple circle; Mean fluorescence intensity of **(B)** FSP1-NF, **(C)** KRT14-NF, **(D)** PECAM1-NF, **(E)** GAPDH-NF from both wound groups (n = 11, duplicate, mean with SEM).

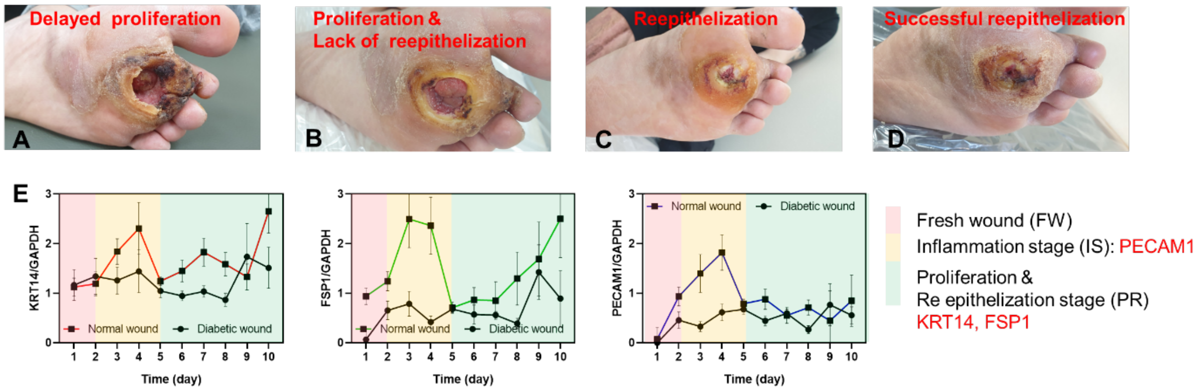


F

| | KRT14 NF | | FSP1 NF | | PECAM1 NF | |
|---|---|---|---|---|---|---|
| | Normal | Diabetic | Normal | Diabetic | Normal | Diabetic |
| Fresh Wound | 1 | 1 | 0-1 | 1 | <1 | <1 |
| Inflammation stage | 1-2.5 | 0-1 | 1-3 | 0-1 | **1-2** | **<1** |
| Proliferation & Reepithelization stage | **1-3** | **1-1.5** | **1-3** | **1-1.5** | <1 | <1 |

**Supplementary Figure S16. A wound healing algorithm for patients with diabetic foot ulcer: (A)** Delayed proliferation stage; **(B)** Proliferative but lack of epithelization stage; **(C)** Starting reepithelization; **(D)** Successful reepithelization; **(E)** Evaluation of target gene expression by NFs on the normal and diabetic groups (target gene/GAPDH) from Figure 5; **(F)** Analyzed wound healing index based on NF's signal from E.
